# A Novel Intra-U1 snRNP Cross-Regulation Mechanism: Alternative Splicing Switch Links U1C and U1-70K Expression

**DOI:** 10.1371/journal.pgen.1003856

**Published:** 2013-10-17

**Authors:** Tanja Dorothe Rösel-Hillgärtner, Lee-Hsueh Hung, Ekaterina Khrameeva, Patrick Le Querrec, Mikhail S. Gelfand, Albrecht Bindereif

**Affiliations:** 1 Institute of Biochemistry, Justus Liebig University of Giessen, Giessen, Germany; 2 Kharkevich Institute for Information Transmission Problems, RAS, Moscow, Russia; 3 Lomonosov Moscow State University, Department of Bioengineering and Bioinformatics, Moscow, Russia; University of California San Francisco, United States of America

## Abstract

The U1 small nuclear ribonucleoprotein (snRNP)-specific U1C protein participates in 5′ splice site recognition and regulation of pre-mRNA splicing. Based on an RNA-Seq analysis in HeLa cells after U1C knockdown, we found a conserved, intra-U1 snRNP cross-regulation that links U1C and U1-70K expression through alternative splicing and U1 snRNP assembly. To investigate the underlying regulatory mechanism, we combined mutational minigene analysis, *in vivo* splice-site blocking by antisense morpholinos, and *in vitro* binding experiments. Alternative splicing of U1-70K pre-mRNA creates the normal (exons 7–8) and a non-productive mRNA isoform, whose balance is determined by U1C protein levels. The non-productive isoform is generated through a U1C-dependent alternative 3′ splice site, which requires an adjacent cluster of regulatory 5′ splice sites and binding of intact U1 snRNPs. As a result of nonsense-mediated decay (NMD) of the non-productive isoform, U1-70K mRNA and protein levels are down-regulated, and U1C incorporation into the U1 snRNP is impaired. U1-70K/U1C-deficient particles are assembled, shifting the alternative splicing balance back towards productive U1-70K splicing, and restoring assembly of intact U1 snRNPs. Taken together, we established a novel feedback regulation that controls U1-70K/U1C homeostasis and ensures correct U1 snRNP assembly and function.

## Author Summary

The accurate removal of intervening sequences (introns) from precursor messenger RNAs (pre-mRNAs) represents an essential step in the expression of most eukaryotic protein-coding genes. Alternative splicing can create from a single primary transcript various mature mRNAs with diverse, sometimes even antagonistic, biological functions. Many human diseases are based on alternative-splicing defects, and most interestingly, certain defects are caused by mutations in general splicing factors that participate in each splicing event. To address the question of how a general splicing factor can regulate alternative splicing events, here we investigated the regulatory role of the U1C protein, a specific component of the U1 small nuclear ribonucleoprotein (snRNP) and important in initial 5′ splice site recognition. Our RNA-Seq analysis demonstrated that U1C affects more than 300 cases of alternative splicing in the human system. One U1C target, U1-70K, appeared to be particularly interesting, because both protein products are components of the U1 snRNP and functionally depend on each other. Analyzing the mechanistic basis of this intra-U1 snRNP cross-regulation, we discovered a U1C-dependent alternative splicing switch in the U1-70K pre-mRNA that regulates U1-70K expression. In sum, this feedback loop controls and links U1C and U1-70K homeostasis to guarantee correct U1 snRNP assembly and function.

## Introduction

In eukaryotes accurate splicing is an essential step in gene expression, because most protein-coding genes contain introns, which have to be removed from the precursor messenger RNA (pre-mRNA) to join the exons to a continuous open-reading-frame. In alternative splicing it is the balance between accuracy and flexibility of splice site recognition that creates from a single transcript multiple isoforms with diverse, sometimes even antagonistic, biological functions [1], [2]. Splice site selection depends on multiple parameters, such as splice site strength, RNA secondary structures, and transcription kinetics, and is modulated by *trans*-acting splicing regulators that can act as activators or repressors.

In both constitutive and alternative splicing, intron removal is catalyzed by the spliceosome, a macromolecular RNA-protein complex that comprises five small nuclear ribonucleoprotein particles (snRNPs) and numerous non-snRNP proteins [3]. Spliceosome assembly is a highly coordinated process characterized by a dynamic RNA-protein network. It is initiated by the recognition of the 5′ splice site by the U1 snRNP, however U1 snRNA:5′ splice site base-pairing alone is not sufficient. This interaction is further stabilized by both U1 snRNP components and non-snRNP factors that contribute to 5′ splice site selection and spliceosome assembly [4]–[6]. In addition to the snRNA, the U1 snRNP contains the Sm protein heptamer and three specific proteins: U1-70K, U1A, and U1C. Besides their role in splicing, both U1A and U1-70K bind directly to the poly(A) polymerase and are thereby involved in U1 snRNP-dependent inhibition of polyadenylation [7]–[9]. This includes the auto-regulation of U1A expression by inhibiting 3′-end processing of its own mRNA [10], [11]. U1-70K and U1C functionally depend on each other: First, the presence of U1-70K is a prerequisite for the stable incorporation of U1C into the U1 snRNP [12], [13]; second, the interaction of U1-70K with SRSF1 (ASF/SF2) stimulates U1 snRNP binding to the 5′ splice site only if U1C is present [14], [15]. Hence, the two proteins strongly rely on each other to ensure correct 5′ splice site recognition by the U1 snRNP.

The U1C protein is particularly important for correct 5′ splice site recognition: Mutational analysis in yeast revealed that U1C is essential for pre-mRNA splicing *in vivo* [16], and U1C stimulates the formation of early splicing complexes by stabilizing the U1 snRNA:5′ splice site duplex [17]–[19]. Consistent with this, structural analyses of the U1 snRNP located U1C in close proximity to the 5′ end of the U1 snRNA and revealed that U1C directly contacts the minor groove of the snRNA:mRNA duplex [20]. Moreover, several studies indicate that U1C participates directly in 5′ splice site choice: Du and Rosbash [21] demonstrated that recombinant yeast U1C protein binds to 5′ splice site consensus sequences independently of the U1 snRNP; moreover, we have recently shown that U1C regulates a subset of alternatively spliced 5′ splice sites in the zebrafish [22].

Here we investigate the role of U1C as an alternative splicing regulator in the human system. Based on an RNA-Seq analysis in HeLa cells after siRNA-mediated knockdown of U1C, we identified a distinct group of target genes with specific U1C-dependent alterations in their splicing patterns. We focus on a particularly interesting target, U1-70K, because these two proteins coexist within the same snRNP and strongly depend on each other (see above). We discovered a conserved, intra-U1 snRNP cross-regulation, the mechanistic basis of which was further investigated, combining mutational minigene analysis, *in vivo* splice-site blocking by antisense morpholinos, and *in vitro* binding experiments. This revealed that recognition of an alternative, U1C-dependent 3′ splice site within intron 7 of the U1-70K pre-mRNA requires binding of intact, U1C-containing U1 snRNPs to downstream cryptic 5′ splice sites. Importantly, this mechanism describes a novel feedback-loop to control U1-70K and U1C homeostasis, linking the expression of these two U1 snRNP-specific factors via alternative splicing.

## Results

### Genome-wide analysis of U1C-dependent alternative splicing in HeLa cells

**Figure 1:**
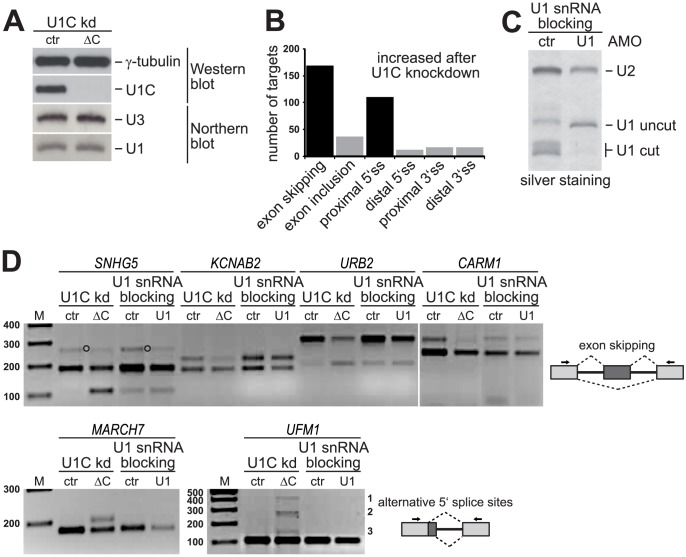
U1C depletion results in specific alternative splicing alterations in HeLa cells: Specificity and validation. (**A**) U1C knockdown (kd) in HeLa cells. Whole cell lysates were analyzed by SDS-PAGE and Western blot detecting U1C and γ-tubulin. U1 snRNA steady-state levels were analyzed by Northern blotting with probes specific for U1 snRNA and, as a loading control, U3 snoRNA. HeLa cells after U1C knockdown (ΔC) and luciferase-siRNA treated control cells (ctr) were compared. (**B**) Graphical overview of U1C-dependent alternative splicing targets identified by RNA-Seq analysis. (**C**) U1 snRNA blocking in HeLa cells. The efficiency of U1 snRNA blocking was determined by RNase H protection and silver staining. The positions of the full-length U1 snRNA (U1 uncut), the RNase H-cleaved U1 snRNA (U1 cut), and the U2 snRNA (as a control) are marked on the right. (**D**) Alternative splicing patterns of selected U1C target genes (names above the lanes) were analyzed by RT-PCR, using total RNA from HeLa cells after U1C-knockdown (ctr vs. ΔC) or U1 snRNA blocking (ctr vs. U1). Target-specific primers (arrows in the schematics on the right of the panels) were designed to amplify both alternative splicing isoforms. *M*, DNA size markers (in bp). Upper panel: Top and lower bands represent exon inclusion and skipping products, respectively; an unspecific product for *SNHG5* is marked by open circles between the lanes. Lower panel: For *MARCH7* top and lower bands reflect usage of the proximal and distal 5′ splice site, respectively. For *UFM1* three alternative 5′ splice sites are activated upon U1C knockdown labeled with 1, 2, and 3 on the right.

To investigate whether U1C plays a splicing-regulatory role in the human system, we performed siRNA-mediated knockdown of U1C in HeLa cells and analyzed alternative splicing patterns by high-throughput RNA sequencing (RNA-Seq). Western blot analysis of whole-cell lysates confirmed that U1C protein is no longer detectable after three days of knockdown in comparison to the control-treated cells (**Figure 1A**). Importantly, as shown by Northern blot analysis of total RNA, U1 snRNA steady-state levels were not affected under U1C knockdown conditions (**Figure 1A**). In addition, affinity purification of U1 snRNPs from both control- and U1C-knockdown cells demonstrated that the U1C-deficient particles are fully stable (**Figure S1A**). *In vitro* binding assays with substrates containing functional 5′ splice sites further showed that the lack of U1C slightly reduces, but does not abolish U1 snRNP binding efficiency (**Figure S1B**).

Deep-sequencing of poly(A)^+^-selected RNA from control- and U1C-siRNA treated HeLa cells yielded 56.9 and 52.0 million 105-bp single-end sequence reads, respectively. 56% for the control sample and 69% for the knockdown could be uniquely mapped to the human genome and annotated splice junctions. Approximately 30% (control: 32%; knockdown: 28%) of the uniquely mapped reads span a splice junction. We applied a data analysis procedure described previously [22] to predict U1C-dependent alternative splicing targets, resulting in these two major alternative splicing changes (summarized in **Figure 1B**):

First, cassette-type exons of which 169 targets were detected with increased exon skipping, and 37 targets with increased exon inclusion upon U1C knockdown (see **Tables S1** and **S2**). Second, we found 111 targets with alternative 5′ splice sites, where usage of the proximal (downstream) site increased upon U1C knockdown, and 12 targets with increased distal (upstream) site usage (see **Tables S3** and **S4**). In addition, there were only 34 cases of alternative 3′ splice sites, 17 of them each with increased usage of the proximal or the distal splice site.

A total of 33 predicted targets with increased exon skipping or increased usage of proximal 5′ splice sites were randomly selected for validation by semi-quantitative RT-PCR: We were able to positively validate 17 out of 19 exon skipping events and 11 out of 14 cases of alternative 5′ splice site usage, corresponding to a general validation rate of ∼85% (**Figure S1C** and **D**).

To further control for the U1C specificity of these alternative splicing changes, we also blocked base pairing between the U1 snRNA and the pre-mRNA 5′ splice site, using an antisense morpholino oligonucleotide (AMO) directed against the 5′ end of the U1 snRNA [23]. Efficient morpholino blocking of the U1 snRNA was confirmed by RNase H protection, using an antisense DNA oligomer binding to the 5′ end of the U1 snRNA and silver staining (**Figure 1C**). Total RNA was then subjected to RT-PCR analysis, using the same target-specific primers used to validate U1C-dependent alternative splicing changes. **Figure 1D** shows six selected targets, four for exon skipping and two for alternative 5′ splice site choice: Knockdown of U1C resulted in increased exon skipping in *SNHG5* (exon 4), *KCNAB2* (exon 3), *URB2* (exon 6), and *CARM1* (exon 15); for other targets, here exemplified by *MARCH7* (exon 7) and *UFM1* (exon 2), an alternative (proximal) 5′ splice site became activated in the absence of U1C. In contrast, the isoform ratio did not significantly change after AMO blocking of U1 snRNA base pairing (**Figure 1D**, compare lanes *U1C kd* and *U1 snRNA blocking*). We note that AMO blocking generally reduced mRNA levels, most likely due to a general splicing block by the AMO treatment. In sum, this direct comparison of the splicing patterns after U1C depletion and after AMO-directed U1 blocking confirmed the U1C specificity of the effects observed.

We conclude that most of U1C-dependent alternative splicing changes fall into two classes, cassette-type exons and alternative 5′ splice site usage. There is a striking bias towards increased exon skipping (169 versus 37), followed by distal-to-proximal 5′ splice site shifts (111 versus 12; **Figure 1B**); therefore U1C appears to play primarily an activating role in 5′ splice site recognition.

### Regulation of U1-70K expression involves U1C-dependent activation of an alternative 3′ splice site

Among the most interesting targets of U1C-dependent alternative splicing we identified the U1-70K pre-mRNA. As described in the Introduction, these two proteins, U1C and U1-70K, are both specific components of the U1 snRNP, interact with each other in the U1 snRNP, and are important for its function in pre-mRNA processing.

**Figure 2:**
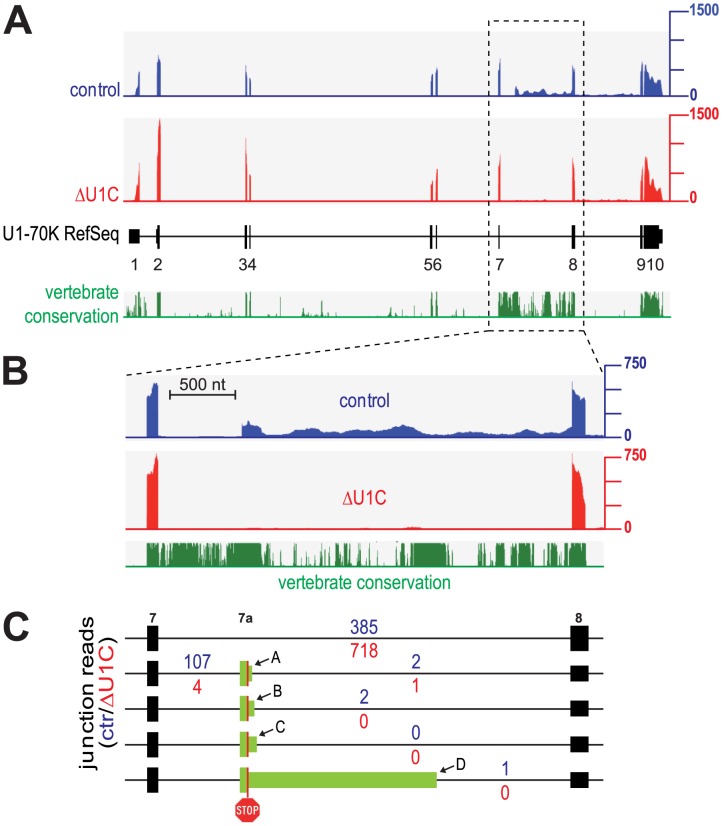
U1C-dependent activation of an alternative 3′ splice site within U1-70K introduces a PTC: RNA-Seq analysis. (**A**) Read-density maps derived from RNA-Seq analysis of control- (blue) and U1C-siRNA-treated (ΔU1C, red) HeLa cells are shown above the exon-intron structure of the human U1-70K gene (exons 1–10 indicated as black boxes, with the narrower parts representing the untranslated regions at the 5′ and 3′ ends). Below, the conservation in vertebrates is given in green. The dashed region is shown in more detail below in panel B. (**B**) Read densities of the control- (blue) and U1C-knockdown HeLa cells (ΔU1C, red) for the U1C-dependent alternatively spliced and highly conserved exons 7–8 region. (**C**) Quantitation of the use of the alternative 3′ splice site in intron 7, as determined by specific junction-read numbers given above and below each exon-intron structure (in blue for control-, in red for U1C-knockdown). The green box indicates the potential alternative exon 7a generated by use of the alternative 3′ splice site at position +642 in intron 7, which introduces a premature termination codon (stop sign), and one of downstream cryptic 5′ splice sites (labeled A, B, C, and D).

**Figure 2A** shows the distribution of read coverage along the U1-70K pre-mRNA (NM_003089) obtained by RNA-Seq analysis of control- and U1C-knockdown HeLa cells. In the wildtype situation (control), we see a significant accumulation of sequence reads in intron 7, starting at position +643 and extending up to the 3′ splice site of exon 8 (**Figure 2A and B**). The RNA-Seq data analysis revealed an alternative 3′ splice site at the position where the intron reads start to accumulate, which is frequently used in comparison to normal, productive exons 7–8 splicing, but strongly depends on U1C (**Figure 2C**; 107 versus 4 junction reads for control and ΔU1C, respectively). Usage of this alternative 3′ splice site introduces a premature termination codon (PTC) into the U1-70K mRNA. Conversely, normal exons 7–8 splicing strongly increases after U1C depletion (718 versus 385 junction reads for ΔU1C and control, respectively). We noted several cryptic 5′ splice sites located closely downstream of the alternative 3′ splice site (referred to as A, B, C), as well as one more further downstream (site D; **Figure 2C**). However, usage of these 5′ splice sites, that means inclusion of the alternative “exon 7a”, is not significant, under both normal and U1C-knockdown conditions. We note that Cunningham et al. [24] had proposed a mechanism by which competing adjacent 5′ splice sites are simultaneously bound by U1 snRNPs and thereby splicing efficiency is reduced because of strong mutual inhibition. Since under normal conditions intron reads in this region are relatively high (**Figure 2B**, control), we conclude that, if the alternative 3′ splice site is used, the remainder of intron 7 remains largely unspliced, and the resulting transcript is expected to be degraded by NMD.

**Figure 3:**
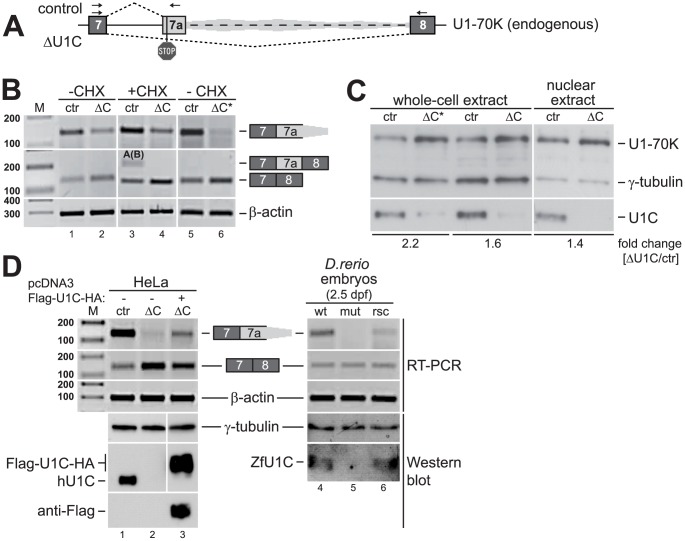
U1-70K mRNA and protein levels are upregulated in human and zebrafish upon loss of U1C. (**A**) Exon-intron structure of U1-70K exons 7–8. The predominant splicing patterns in control and in U1C-knockdown HeLa cells are depicted by dashed lines above and below the schematic, respectively. The grey shading downstream of “exon 7a” indicates that the 7a-8 junction is not detectable in control cells without cycloheximide treatment. Arrows represent the RT-PCR primers used in (B) and (D) to detect alternative 3′ splice site activation (primers 7-7a), exon 7a inclusion, and exons 7–8 splicing. (**B**) U1-70K alternative splicing after U1C knockdown. HeLa cells were treated with two different siRNAs against U1C (ΔC or ΔC*, see Materials and Methods), or a control siRNA (ctr), comparing untreated cells (−CHX) or cells after treatment with cycloheximide (+CHX). Alternative splicing patterns were analyzed by RT-PCR. The label within the middle panel indicates which cryptic 5′ splice sites are used for exon7a inclusion, with letters in parentheses marking the less frequently used splice sites (see **Figure S3A**). β-actin serves as an internal loading control. (**C**) U1-70K protein levels after U1C knockdown in HeLa cells. Whole cell lysates or nuclear extracts were analyzed by SDS-PAGE and Western blot, detecting U1-70K, γ-tubulin (as a loading control), and U1C. The fold change of U1-70K protein expression after U1C knockdown is given below (ΔU1C/ctr). (**D**) Add-back of U1C restores normal U1-70K splicing in HeLa and zebrafish (*D. rerio*). Left panel: Control- (ctr) and U1C-knockdown (ΔC) HeLa cells are compared with U1C-knockdown cells expressing Flag/HA-tagged U1C. Knockdown and over-expression were verified by Western blot analysis. Right panel: A wildtype zebrafish embryo (wt) is compared with two U1C-knockout mutants, without (mut) and with ZfU1C-cRNA injection (rsc = rescue). In both assays, alternative splicing patterns were analyzed by RT-PCR, and β-actin serves as an internal loading control. The rescue effect was confirmed by Western blot analysis of single-embryo lysates. M, DNA size markers (in bp).

To validate our RNA-Seq data and to assess the NMD effect, alternative splicing of the U1-70K exons 7–8 region was analyzed by RT-PCR, using total RNA from control- and U1C-knockdown HeLa cells (**Figure 3A**). Specific primers were located in the constitutive exons 7 and 8, as well as immediately downstream of the predicted PTC, but upstream of the cryptic 5′ splice sites. Knockdown of U1C decreased recognition of the alternative 3′ splice site in intron 7; conversely, more functional U1-70K mRNA was produced as shown by the increase of the spliced exons 7–8 product (**Figure 3B**, lanes 1/2 and 5/6). Off-target effects were ruled out by comparing two different U1C-specific siRNAs (one located in the 3′ UTR and another one within the open-reading-frame; U1C vs. U1C*). To test whether exon 7a inclusion indeed results in NMD, the cells were additionally treated with cycloheximide for 5 hours (after three days of siRNA treatment) to block translation and thereby NMD (**Figure 3B**, lanes 3 and 4). We were able to detect exon 7a inclusion under these conditions using a primer pair located in exons 7 and 8; sequencing and analysis of the RT-PCR products by Bioanalyzer revealed that predominantly the cryptic 5′ splice sites A and B were used, with site A being more frequently used than site B; usage of 5′ splice site C, however, was not significant (for a detailed analysis of splice site usage, see **Figure S3A**). Notably, sites A and B are much weaker 5′ splice sites than site C (splice site scores: 5.29, 3.38, and 8.91, respectively [25]). Since we can detect exon 7a inclusion only after cycloheximide treatment, we conclude that activation of the alternative 3′ splice site of exon 7a does activate NMD, thereby efficiently removing the non-productive splice isoform of U1-70K.

In addition, after validating these effects on the U1-70K mRNA, we assayed for up-regulation of the U1-70K protein (**Figure 3C**). Western blot analysis of both whole-cell and nuclear extracts from HeLa cells after U1C knockdown demonstrated that indeed the U1-70K protein levels correlate with the mRNA levels: Upon loss of U1C we detected an increase of U1-70K protein (between 1.4-fold in nuclear extract and 1.6-/2.2-fold in whole-cell extract).

To confirm the U1C specificity of the alternative splicing changes described above, we combined knockdown of endogenous U1C expression and over-expression of Flag/HA-tagged U1C in HeLa cells (**Figure 3D**, left panel). Clearly, add-back of FLAG/HA-tagged U1C increased exons 7-7a splicing, although control levels were not completely restored; this may be due to inefficient U1 snRNP incorporation or function of the FLAG/HA-tagged U1C protein. At the same time normal exons 7–8 splicing decreased.

Taken together, we conclude that this unusual alternative splicing regulation of U1-70K expression specifically depends on U1C.

### Conservation of the U1C-dependent alternative splicing switch of U1-70K expression

Intron 7 contains highly conserved regions, in particular the first 0.8 kb, which include the alternative 3′ splice site and the cryptic 5′ splice sites (**Figure 2B** and **Figure S2A**). Therefore, we investigated the conservation of the U1C-dependent effects observed on U1-70K alternative splicing in zebrafish and mouse. First, we used a zebrafish U1C knockout mutant and performed *in vivo* rescue as previously established [22]. In brief, *in vitro* transcribed ZfU1C cRNA was injected into U1C mutant zebrafish embryos at the one-cell stage. 2.5 days-post-fertilization rescued embryos were selected according to their phenotypic appearance, and restoration of ZfU1C protein expression was confirmed by Western blotting (**Figure 3D**, right panel). RT-PCR analysis of total RNA from single embryos showed that U1C knockout in zebrafish completely abolished exon 7a inclusion (**Figure 3D**, top panel: compare lanes 4 and 5). Add-back of U1C (rescued individual) reactivated the alternative 3′ splice site of exon 7a (7-7a; **Figure 3D**, top panel lane 6). Second, we performed siRNA-mediated knockdown of U1C in mouse myoblast cells (**Figure S2B**) and detected a strong decrease in exon 7a inclusion after U1C depletion. In summary, the alternative splicing switch of U1-70K expression appears to be conserved among different vertebrates.

### Activation of the alternative 3′ splice site requires downstream cryptic 5′ splice sites and their recognition by U1 snRNPs

**Figure 4:**
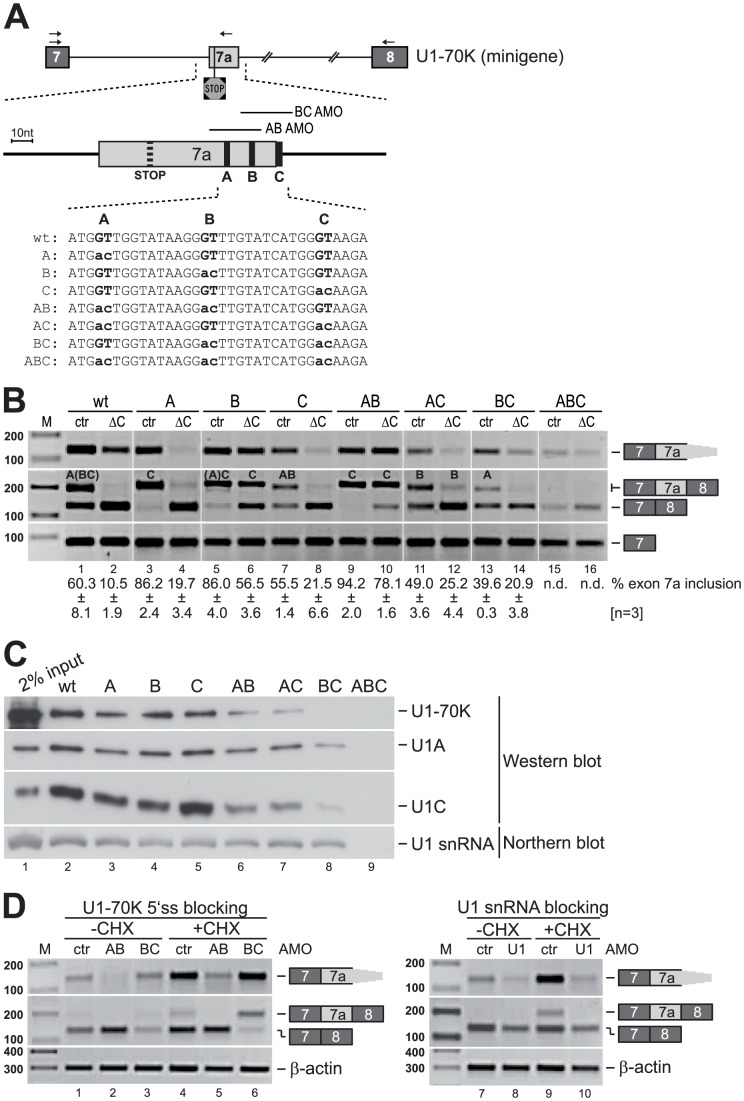
Alternative 3′ splice site activation requires U1 snRNP binding to downstream cryptic 5′ splice sites. (**A**) U1-70K minigene constructs used for *in vivo* splicing analysis (see panel B), including exons 7, 7a, and 8; the arrows indicate the primers used for RT-PCR analysis. The enlargement below represents the region covered by the biotinylated transcripts used for *in vitro* binding studies (see panel C). The exact positions of the stop codon (dashed vertical line) and the three cryptic 5′ splice sites (bold vertical lines labeled with A, B, and C) are shown together with the 5′ splice site sequences, including all point mutations analyzed. The two solid lines above the enlarged exon mark the positions of the splice site blocking antisense morpholinos (see panel D); their labeling (AB and BC AMO) refers to the 5′ splice site they block. (**B**) Mutational analysis of U1-70K alternative splicing. Splicing patterns of U1-70K minigenes (as indicated) in control- (ctr) and U1C-knockdown (ΔC) HeLa cells were analyzed by RT-PCR, detecting alternative 3′ splice site activation (primers 7-7a; top panel), exon 7a inclusion and skipping (primers 7–8; middle panel), and as a loading control, exon 7 alone (bottom panel); splicing products are depicted on the right. Percentages of exon 7a inclusion are given below with standard deviations calculated from three individual experiments [n = 3]; the labels within the second panel indicate, which cryptic 5′ splice sites were used in each case for 7a inclusion, with letters in parentheses marking the less frequently used splice sites (see **Figure S3B**). (**C**) U1 snRNP binds to cryptic 5′ splice sites of U1-70K exon 7a. 3′-biotinylated RNAs spanning “exon 7a” including flanking intronic sequences (as shown enlarged in the middle of the schematic in panel A) were incubated with HeLa nuclear extracts (2% input). Bound proteins (2/3 of selected material) were analyzed by Western blotting, using antibodies against U1-70K, U1A, and U1C; bound U1 snRNA was detected by Northern blot hybridization. (**D**) Antisense morpholino (AMO) transfection in HeLa cells. In two separate assays HeLa cells were transfected with AMOs either against the cryptic 5′ splice sites of U1-70K exon 7a (AB, BC; left panel) or the 5′ end of U1 snRNA (U1; right panel), or with an unspecific control morpholino (ctr). Cells were either left untreated (−CHX) or were treated with cycloheximide (+CHX) before RT-PCR analysis. The label within the middle panel indicates which cryptic 5′ splice sites are used for exon7a inclusion, with letters in parantheses marking the less frequently used splice sites. M, DNA size markers (in bp).

In view of the known functions of U1C in 5′ splice site choice, it was rather unexpected to discover a case of U1C-dependent activation of a 3′ splice site. In order to study the mechanistic basis of this unusual regulation in more detail, we next examined whether the cryptic 5′ splice sites identified downstream of the alternative 3′ splice site are important for its activation. Minigene constructs of U1-70K exons 7 to 8 were generated, maintaining the most highly conserved regions in intron 7 (see **Figure 2B**), including the alternative 3′ splice site and the three cryptic 5′ splice sites downstream (called A, B, and C; see **Figure 4A**). Point mutations were introduced to inactivate the cryptic 5′ splice sites individually, in combinations of two, and all three of them (**Figure 4A**). To analyze the U1C-dependent splicing patterns of the different minigenes *in vivo*, they were transfected into HeLa cells after three days of U1C or control knockdown; 24 hours later total RNA was isolated for RT-PCR analysis (**Figure 4B**). In contrast to the endogenous U1-70K expression, where NMD is active, this minigene analysis allowed monitoring the activation of the alternative 3′ splice site by measuring exon 7a inclusion.

As we had observed for the endogenous U1-70K gene (see above), the alternative 3′ splice site was also in this minigene context partially used, so that both exon 7a inclusion and skipping isoforms were detectable. For the wildtype minigene, splicing consistently occurred through 5′ splice site A, and only to a minor extent through sites B and and C. After U1C knockdown exon 7a skipping strongly increased, reproducing the U1C-dependent use of the alternative 3′ splice site (**Figure 4B**, lanes 1 and 2).

Mutating 5′ splice sites A, B, or both in combination strongly increased exon 7a inclusion in the presence of U1C (A, B, and AB mutants: **Figure 4B**, compare lanes 1 with 3, 5, and 9); splicing used almost exclusively 5′ splice site C. Interestingly, mutants B and AB showed strong exon 7a inclusion even in the absence of U1C (lanes 6 and 10). After mutation of 5′ splice site C alone, or in combination with A or B, both exon 7a skipping and inclusion were detected (C, AC, and BC mutants: compare lanes 1 with 7, 11, and 13); after U1C knockdown those mutants showed no significant exon 7a inclusion. Only when all three sites were inactivated (mutant ABC: lanes 15 and 16), complete skipping resulted both in the presence and absence of U1C (for a detailed analysis of splice site usage, see **Figure S3B**).

Together this indicates that the three regulatory 5′ splice sites are particularly important for modulating the use of the exon 7a 3′ splice site in the context of minigene construct, maintaining the balance between skipping and inclusion and sensing U1C-containing versus -deficient U1 snRNPs. Specifically, the splice sites show a differential requirement for U1C in 3′ splice site activation: Splice sites A and B negatively regulate 3′ splice site activation and strongly depend on U1C. In contrast, 5′ splice site C acts positively and appears to be U1C-independent. Therefore we conclude there is a complex interaction network of the three 5′ splice sites, with the three sites contributing both positive and negative individual effects and differential U1C sensitivity.

Next, to investigate U1 snRNP binding to the cryptic 5′ splice sites, we carried out *in vitro* binding assays. Short RNAs (139 nt) spanning the exon 7a region of U1-70K (enlargement in schematic of **Figure 4A**) were incubated in HeLa nuclear extract, comparing the wildtype sequence and derivatives with the mutated cryptic 5′ splice sites described above. Western and Northern blot analyses of bound proteins (U1-70K, U1A, U1C) and U1 snRNA, respectively, showed that mutation of 5′ splice sites A or B alone slightly reduced U1 snRNP binding (**Figure 4C**, compare lanes 3–5); in contrast, mutating splice site C alone showed the same pulldown efficiency as the wildtype sequence (**Figure 4C**, lanes 1 and 5). However, all double-mutants (AB, AC, and BC) strongly reduced, and the triple-mutant (ABC) completely lost U1 snRNP binding capacity (**Figure 4C**, lanes 6 to 9). In sum, this suggests an additive behavior of the three cryptic 5′ splice sites in U1 snRNP binding.

We also studied in the endogenous context of the U1-70K gene how important U1 snRNP binding to the cryptic 5′ splice sites is for 3′ splice site activation, using *in vivo* splice site blocking experiments (**Figure 4D**). Antisense-morpholinos directed against the three cryptic 5′ splice sites were transfected into HeLa cells, blocking either sites A and B together or B and C (**Figure 4D**, lanes 1–3). In addition, cells were treated with cycloheximide to inhibit NMD and thereby stabilize RNAs where the exon 7a 3′ splice site had been used (lanes 4–6). Blocking of 5′ splice sites A and B (AB) strongly reduced 3′ splice site activation (spliced exons 7-7a), and exon 7a inclusion (7-7a-8) was undetectable (**Figure 4D**, compare lanes 1/2 and 4/5). In contrast, blocking 5′ splice sites B and C (BC) still allowed efficient recognition of the alternative 3′ splice site and – under cycloheximide conditions – exon 7a inclusion (**Figure 4D**, lanes 3 and 6). We conclude that also in the endogenous context 5′ splice sites A and B appear to be involved in and splice site A to be sufficient for 3′ splice site activation.

Surprisingly, AMO blocking of the two 5′ splice sites A and B inhibited 3′ splice site activation *in vivo*, whereas mutating sites A and/or B in the minigene resulted in strong exon 7a inclusion. Note that in the endogenous 70K gene the 5′ splice site C was not used (**Figure 3B**, lane 3); we assume that in the minigene context the 5′ splice site C-mediated 3′ splice site activation is favoured because of the construct design, in which the intronic sequence between 5′ splice site C and the downstream 3′ splice site was shortened dramatically. Thus, exon 7a inclusion using site C in the minigene may be induced by intron definition rather than by exon definition mechanism that occurs when the regulatory 5′ splice sites A and B are active.

Finally, we addressed the question whether base-pairing of the U1 snRNA is essential for 3′ splice site activation: HeLa cells were transfected with an AMO that blocks the 5′ end of the U1 snRNA to generally inhibit U1 snRNP binding (for a control of AMO binding to the U1 snRNA see **Figure 1B**. Again, exon 7a inclusion was observed only in cells that had been treated with the control morpholino under cycloheximide conditions (**Figure 4D**, right panel). However, inhibition of U1 snRNP binding nearly abolished recognition of the alternative 3′ splice site and resulted in efficient skipping of exon 7a. The minor decrease observed for U1-70K exons 7–8 splicing as well as for the β-actin control probably reflects the general splicing inhibition by the U1 snRNP blocking oligonucleotide. However, U1-70K exons 7-7a splicing is clearly much more severely affected by U1 snRNP blocking, most likely due to differential stability and turnover of these spliced products. In summary, our *in vitro* binding experiments demonstrated that a direct interaction between U1 snRNA and the cryptic 5′ splice sites is necessary but without U1C not sufficient for 3′ splice site activation.

### U1-70K knockdown results in co-depletion of U1C protein and in U1-70K/U1C-double-deficient U1 snRNPs

**Figure 5:**
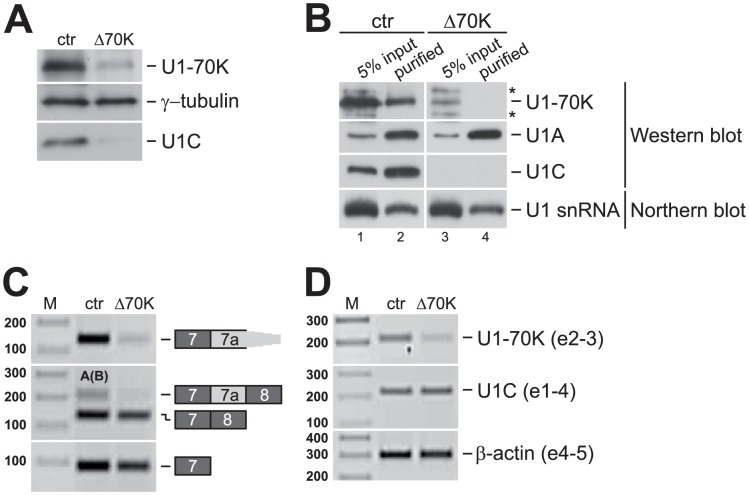
U1-70K knockdown results in co-depletion of U1C protein and in U1-70K/U1C double-deficient U1 snRNPs. (**A–B**) 96 hours after siRNA transfection, whole-cell lysates were prepared from control- (ctr) and U1-70K-knockdown- (Δ70K) cells. (**A**) U1-70K knockdown in HeLa cells results in co-depletion of U1C. Whole-cell lysates were analyzed by SDS-PAGE and Western blot, detecting U1-70K, γ-tubulin, and U1C. (**B**) U1-70K/U1C double-deficient U1 snRNPs in U1-70K-knockdown HeLa cells. U1 snRNPs were affinity-purified from whole-cell lysates (5% of input, lanes 1 and 3; 2/3 of purified material, lanes 2 and 4) and analyzed by SDS-PAGE and Western blot, detecting U1-70K, U1A, and U1C. U1 snRNA was detected by Northern blotting, analyzing 1/3 of the purified material. Asterisks mark unspecific bands detected by the anti-U1-70K antibody. (**C**) Exon 7a skipping upon U1-70K knockdown. 72 hours after siRNA transfection, the U1-70K wildtype minigene construct (see **Figure 4A**) was transfected into HeLa cells, and 24 hours later minigene splicing patterns were analyzed by RT-PCR, comparing control- (ctr) and U1-70K- (Δ70K) knockdown samples. Specific primer sets were used to detect alternative 3′ splice site activation (product 7-7a), exon 7a inclusion (product 7-7a-8; letters indicate which cryptic 5′ splice sites are used, with parentheses marking the less frequently used splice site; see **Figure S3A**), exon 7a skipping (product 7–8), and as a loading control, exon 7 alone (product 7). The identities of the splicing products are depicted on the right. (**D**) U1C mRNA is stable after U1-70K knockdown. After four days of U1-70K knockdown in HeLa cells, total RNA was isolated from control- (ctr) and U1-70K- (Δ70K) knockdown cells. Endogenous mRNA levels of U1-70K, U1C, and β-actin (as indicated on the right) were analyzed by RT-PCR. The numbers given in parentheses refer to the amplified exons. M, DNA size markers (in bp).

After we had established that U1-70K levels are regulated by a U1C-dependent alternative splicing event, we asked whether a reciprocal reduction of U1-70K protein may affect U1C protein levels and/or the molecular composition of the U1 snRNP. We performed siRNA-mediated knockdown of U1-70K in HeLa cells and discovered that along with U1-70K protein, U1C protein levels were also strongly reduced in comparison to the control-treated cells (**Figure 5A**). Furthermore, affinity purification of the U1 snRNP from control- and U1-70K-knockdown cells confirmed that the U1 snRNP lacks both proteins (**Figure 5B**). However, U1A remained stably bound to the U1 snRNA, which itself appeared to be unaffected by the loss of U1-70K and U1C.

In order to investigate whether this U1-70K/U1C-deficient U1 snRNP has the same effect on U1-70K alternative splicing as observed for the U1C-deficient particle, we performed *in vivo* splicing assays in control- and U1-70K-knockdown HeLa cells, using our U1-70K wildtype minigene (as described above). RT-PCR analysis showed that exon 7a inclusion was hardly detectable after U1-70K knockdown (**Figure 5C**). These observations are clearly consistent with a U1C-dependent regulation of U1-70K alternative splicing: Loss of U1-70K reduces levels of total and U1 snRNP-bound U1C, which in turn shifts the alternative splicing balance from the non-productive (exons 7-7a) towards the productive isoform (exons 7–8), increasing U1-70K protein levels.

To address the question how U1-70K reduction resulted in U1C depletion, we examined U1C mRNA stability and potential alternative splicing of U1C pre-mRNA, using RT-PCR and total RNA from control- and U1-70K-knockdown HeLa cells. **Figure 5D** shows that U1C mRNA levels remained unchanged after U1-70K knockdown (detecting U1C exons 1–4, middle panel). We can also rule out that an annotated alternatively spliced mRNA isoform of U1C without exon 2 is produced (NR_029472), which would introduce a PTC and should therefore reduce mRNA and protein levels. We did neither see a change in the intensity of the exons 1–4 band (212 bp), comparing control- and U1-70K-knockdown samples, nor could we detect the exon 2 skipping product (169 bp). In conclusion, since U1C mRNA stability and alternative splicing appear not to be affected by U1-70K knockdown, the down-regulation of the U1C protein levels most likely occurs on the level of protein stability and/or translation.

## Discussion

Alternative splicing can produce various mRNA isoforms from one precursor transcript by modulating splice site usage in a tissue or developmental-specific manner [26]. In general, alternative splicing factors, such as SR- or hnRNP proteins, are responsible for regulated activation or repression of certain splicing signals [27], [28]. However, the availability of general splicing factors can also influence alternative splicing events as described [22], [29]–[31; this work].

Here we have investigated the regulatory role of U1C in the human system, and found that U1C knockdown in HeLa cells leads to specific alternative splicing alterations rather than a general block of splicing activity. Thus, our analysis gives a genome-wide overview on how a general snRNP protein, which participates in each splicing reaction, can regulate alternative splicing. The two main alternative splicing modes we identified after U1C depletion were increased exon skipping and changes in the usage of alternative 5′ splice sites. Weakly defined exons more strongly depend on accurate U1 snRNP binding and therefore loss of U1C, which generally promotes correct 5′ splice site recognition, is expected to induce exon skipping. In the case of competing 5′ splice sites, it is known that although they can be bound simultaneously by separate U1 snRNPs the downstream one is preferentially used for splicing [32]. In addition to factors known to regulate the choice between distal and proximal 5′ splice sites (such as hnRNP A1 and its antagonist SRSF1) [33]–[35], we have identified here U1C, which appears to be important to promote splicing at the upstream site, consistent with our earlier study in zebrafish [22]. Although we do not know the mechanistic basis of the U1C dependency, this allows the cell to respond to variable U1 snRNP levels by changes in alternative splicing patterns. In sum, our results demonstrate that U1C acts primarily as a splicing activator.

Since U1C is known to stabilize base-pairing between the U1 snRNA and the 5′ splice site, loss of U1C would be expected to impair general splicing activity. However, we found a distinct group of target genes that are affected rather than a general block of splicing activity. Additionally, most targets do not change their U1C-dependent alternative splicing patterns upon U1 snRNP blocking via antisense morpholinos. Therefore we suggest there are target-specific requirements that determine the level of U1C dependency in each case. In fact, our results raise the question, whether U1C is at all a “general” splicing factor or rather a spliceosome-associated alternative splicing regulator.

One U1C target, U1-70K, appeared to be particularly interesting: First, U1-70K and U1C proteins physically interact with each other in the U1 snRNP, with U1C incorporation depending on prior U1-70K binding to loop I of the U1 snRNA. Second, considering the predominant role of U1C in 5′ splice site recognition, it was surprising to discover a case of U1C-dependent 3′ splice site activation. Therefore we decided to analyze in more detail the mechanistic basis for this unusual intra-U1 snRNP cross-regulation. Our RNA-Seq analysis in combination with RT-PCR validation had revealed that under normal conditions an alternative 3′ splice site within intron 7 of the U1-70K pre-mRNA is frequently used, which introduces a PTC into the mRNA and thereby is expected to induce NMD. Previous work had reported for several alternative splicing factors and core splicesosomal components auto-regulatory feedback mechanisms that involve alternative splicing and NMD (for example, [36]–[38]). In contrast to that, however, we found that U1-70K alternative splicing regulation strongly depends on the presence of U1C, and therefore we propose an intra-U1 snRNP cross-regulation mechanism (see below).

What are the sequence requirements for this U1C-dependent alternative splicing process? Downstream of the U1C-dependent alternative 3′ splice site several cryptic 5′ splice sites are located, that turned out to be critical for the regulation we observed. Under normal conditions, those 5′ splice sites are only very rarely used for splicing (only detectable in the absence of NMD), most likely because of their close proximity to each other. Our *in vitro* binding and *in vivo* antisense-morpholino blocking experiments confirmed that these regulatory 5′ splice sites efficiently bind U1 snRNPs and that this interaction requires base-pairing with the 5′ end of U1 snRNA.

Mutational analysis of these regulatory 5′ splice sites demonstrated that only two of them (labeled A and B in **Figure 2C**) convey U1C dependency, with one of them being sufficient for 3′ splice site activation. In fact, these two sites have very low splice site scores (5.29 and 3.38 for site A and B, respectively, compared to 8.91 for site C), which may explain the stringent requirement for U1C to promote U1 snRNP binding to these weak 5′ splice sites. In addition, when analyzed separately by mutant minigenes (see Results and Figure 4B), sites A/B and C behave as negative and positive regulatory elements, respectively, suggesting an intricate network of these regulatory elements, sensing U1C-containing and –deficient U1 snRNPs. This may enhance U1 snRNP binding for efficient 3′ splice site activation, and second, it may contribute to rapid responsiveness and fine-tuning of the regulatory mechanism.

We noted that the sequence including the alternative 3′ splice site and the regulatory 5′ splice sites downstream are highly conserved among vertebrates (**Figure S2A**). Accordingly, we demonstrated that in zebrafish embryos as well as in C2C12 mouse cells usage of the alternative 3′ splice site and exons 7-7a splicing was strongly reduced after U1C depletion, indicating that the entire U1C/U1-70K cross-regulatory mechanism is conserved.

**Figure 6:**
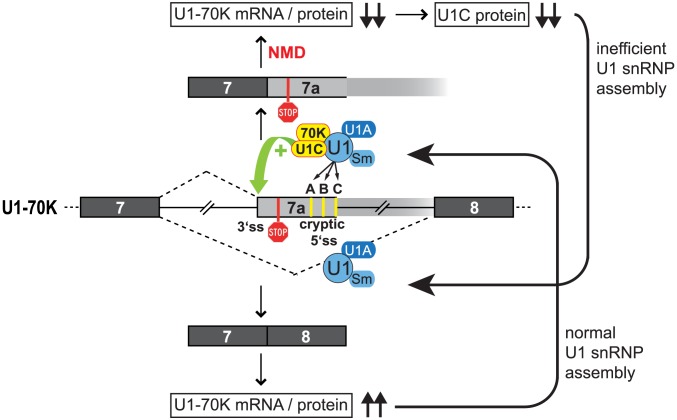
Model of intra-U1 snRNP U1-70K/U1C cross-regulation. Binding of intact U1 snRNPs to three cryptic 5′ splice sites (labeled with A, B, C) within intron 7 of the U1-70K pre-mRNA (middle) activates an alternative 3′ splice site. Inclusion of the alternative exon 7a introduces a premature termination codon (stop sign) into the mature U1-70K mRNA, which is degraded by nonsense-mediated decay (NMD; following the pathway upwards). Reduced U1-70K mRNA and protein levels result in a co-depletion of U1C protein; thus, U1 snRNPs are assembled inefficiently. U1C/U1-70K-deficient U1 snRNPs are unable to activate the alternative 3′ splice site, therefore, constitutive U1-70K splicing is enhanced, and more functional U1-70K mRNA and protein are produced (following the pathway downwards). Normal U1 snRNP assembly is restored and alternative 3′ splice site activation can occur again to close the regulatory circle (for a detailed description, see *Discussion*).

In sum we established the following model of U1-70K/U1C cross-regulation (**Figure 6**): Alternative splicing of the U1-70K pre-mRNA provides the central switch between a productive (exons 7–8) and a non-productive (exons 7-7a) splicing mode, whose balance is determined by U1C and U1-70K protein levels. If U1-70K mRNA and protein levels decrease, U1C is co-depleted and U1 snRNPs are assembled inefficiently. This co-depletion of U1C appears to be mediated on the protein level; for example, free U1C protein, which is not assembled into U1 snRNPs may be less stable (**Figure 5A and B**). The same disturbance of balanced U1-70K alternative splicing can be initiated by U1C knockdown, resulting in U1 snRNPs defective only in U1C. Neither U1C- nor U1C/U1-70K-defective U1 snRNPs are unable to activate the alternative 3′ splice site in U1-70K intron 7, shifting the balance towards productive U1-70K exons 7–8 splicing. As a result, more functional U1-70K mRNA and protein are produced, restoring normal U1 snRNP assembly. Binding of intact, U1 snRNPs to the regulatory 5′ splice sites activates again the U1-70K alternative 3′ splice site and exon 7a inclusion, thereby shifting the alternative splicing balance back towards the non-productive, NMD-inducing mode. The resulting reduction in U1-70K mRNA and protein levels closes the circle. At this point, we cannot rule out a more direct function of U1-70K in the regulation of its own expression, because the effects of U1-70K depletion alone cannot be tested. Mechanistically, the switch could be triggered by a failure of U1-70K to cooperate with SRSF1 to efficiently activate the cryptic 5′ splice sites, which in turn would activate the alternative 3′ splice site through an exon-definition complex. Thus U1-70K itself would be the direct trigger for the alternative splicing switch, and U1C depletion would mimic a lack of U1-70K, because U1-70K is not able to efficiently interact with SRSF1 in the absence of U1C [14], [15]. This situation is reminescent of the auto-regulatory feedback mechanism described for the minor-spliceosomal U11 snRNP: As described by Verbeeren et al. [39], the U11 snRNP can bind to tandem regulatory 5′ splice sites of the minor type, which activates a 3′ splice site in the pre-mRNAs for U11-48K and U11/U12-65K proteins. In contrast, we describe here that one particular component of the U1 snRNP, U1C, is necessary for efficient activation of a 3′ splice site within U1-70K intron 7, and that this is part of a regulatory circuit linking the expression of both U1C and U1-70K proteins. Notably, U11-48K has a similar role in 5′ splice site recognition for the minor spliceosome as U1C for the major spliceosome [39], [40]. Thus, both spliceosomes appear to regulate the expression of their intrinsic factors by a comparable mechanism to ensure correct 5′ splice site recognition.

Taken together we describe a novel and conserved intra-U1 snRNP cross-regulation mechanism that ensures U1-70K and U1C homeostasis and guarantees stoichiometrically correct U1 snRNP assembly. This provides a new paradigm for and mechanistic insight in molecular communication within the spliceosome. It opens up another emerging new question of wide general interest, how the biosynthesis of the more than 100 protein and RNA components of the spliceosome is coordinated.

## Materials and Methods

### siRNA knockdown, antisense morpholino transfection, and cycloheximide treatment

siRNA duplexes were transfected into HeLa cells using Lipofectamine 2000 (Invitrogen) according to the manufacturer' instructions. For U1C knockdown, siRNAs specific for the human U1C mRNA located in the 3′ UTR (ΔC: 5′-AGGCCUUAUUGUAUCGGUU[dT][dT]) or the open-reading-frame (ΔC*: AAACAACGGCUGCAUUUCAAC[dA][dC]) were transfected at a final concentration of 40 nM. For U1-70K knockdown, a specific siRNA (ΔU1-70K: 5′- GAGAGGAAAAGACGGGAAA[dT][dT]) was transfected at a final concentration of 60 nM. For U1C knockdown in C2C12 cells, an siRNA against the mouse U1C mRNA was reverse transfected (at a final concentration of 140 pmol), using Lipofectamine RNAiMax Reagent (Invitrogen). An siRNA specific for the firefly luciferase mRNA (5′-CGUACGCGGAAUACUUCGA[dT][dT]) served as a control (ctr) in all cases. Knockdown efficiencies were determined by Western blot, using monoclonal antibodies against U1C (4H12, Santa Cruz), U1-70K (H111, Synaptic Systems), and, as a control, γ-tubulin (GTU-88, Sigma).

U1 snRNA 5′ end and U1-70K cryptic 5′ splice site blocking were achieved by antisense morpholino transfections as described elsewhere [22]: Briefly, transfections were performed, using the Nucleofector Solution R (Lonza) and the Nucleofector Programm I-013 according to the manufacturer's instructions. 1.5×10^6^ HeLa cells were transfected with AMO (U1, 5′-GGTATCTCCCCTGCCAGGTAAGTAT-3′, at 100 µM [23]; AB, 5′-ACAAACCCTTATACCAACCATACAC-3′ and AC, 5′- GATCTTACCCATGATACAAACCCTT-3′, at 50 µM each), and 15 hours later total RNA was isolated for further analysis. A control AMO (ctr, 5′-CCTCTTACCTCAGTTACAATTTATA-3′, [23]) was transfected at the same concentrations as used for the specific AMO. The efficiency of U1 snRNA inhibition was analyzed by an RNase H protection assay: Whole cell extracts were incubated with 5 µM antisense DNA oligonucleotide (5′-CAGGTAAGTAT-3′) and 1.5 U RNase H (Promega) for 30 min at 37°C. After phenol extraction the total RNA was analyzed on a 10% denaturing polyacrylamide gel followed by silver staining.

For NMD inhibition the growth medium was supplemented with 50 µg/ml cycloheximide (+CHX), and cells were harvested 5 hours later for Western blot analysis and total RNA isolation.

### U1C expression construct and add-back

For the expression construct the coding region of U1C was amplified from cDNA generated from HeLa total RNA. The N-terminal Flag- and the C-terminal HA-tags were introduced by PCR, followed by cloning into pcDNA3 (Invitrogen) between the *Hin*dIII and *Xho*I restriction sites.

For simultaneous U1C over-expression and knockdown of endogenous protein, first, the U1C expression vector (pcDNA3_Flag-U1C-HA) was transfected (TurboFect *in vitro* Transfection Reagent, Fermentas), and 24 hours later siRNAs (final concentration: 140 pmol) were reverse-transfected, using Lipofectamine RNAiMax Reagent (Invitrogen). Overexpression was detected by Western blot, using monoclonal antibodies against U1C (4H12, Santa Cruz) and Flag (Sigma).

### RNA-Seq sample preparation, target gene selection, and RT-PCR validation

For details on poly(A)^+^-RNA selection for Solexa high-throughput sequencing (GAIIx), the data analysis, and alternative splicing target selection, see Rösel et al. [22]. Total RNA from HeLa cells was prepared 72 hours after siRNA transfection by TRIzol reagent (Invitrogen) and RNeasy kit (QIAGEN). The 105-bp single-end sequence reads were aligned to human genome (hg19) and a junction sequence data constructed with Gene Annotations from ENCODE Version 11. RNA-Seq raw data and processed coverage data were uploaded to the GEO database at NCBI (GSE42485).

For target gene validation, 1 µg total RNA was reverse transcribed (iScript cDNA synthesis kit, BioRad) and subjected to PCR using specific primer sets that span the region of interest. **Table S5** lists all oligonucleotides used.

### *In vivo* U1-70K minigene splicing analysis

Three days after siRNA transfection, U1-70K minigene constructs (5 µg per 6-cm dish) were transfected into HeLa cells using FuGeneHD (Promega), and 24 hours later total RNA was isolated using Trizol (Invitrogen) and treated with RQ1-DNase (Promega). Reverse transcription was performed using the minigene specific *BGHreverse* primer (qScript Flex cDNA Kit; Quanta Biosciences), and for PCR gene-specific primers were used to analyze alternative splicing patterns.

The wildtype minigene construct was amplified in several PCR steps from HeLa genomic DNA and cloned into pcDNA3 vector using *Bam*HI and *Eco*RI restriction sites. The final three-exon-construct (7-7a-8) comprises the full sequence of U1-70K exon 7, intron 7 positions 1–844, 1,928–2,227, and 3,077–3,162, with the sequences in between deleted (1,083 nt and 849 nt), and the full sequence of exon 8. Based on the wildtype construct point mutations were introduced by PCR, substituting the GT of the cryptic 5′ splice sites by AC (**Figure 4A**). Only the second half of the construct was reamplified to insert the point mutation and cloned into the wildtype construct, using an endogenous *Xcm*I restriction site and the *Eco*RI site introduced with the exon 8 reverse primer.

Ethidium bromide-stained bands were quantified using the GeneTools software provided with the G:BOX gel documentation system from SynGene.

For better resolution exon 7a inclusion products (primers from exon 7–8), 1 µl of selected reactions (**Figure S3**) were analyzed on an Agilent 2100 Bioanalyzer DNA 1000 Chip.

### *In vitro* binding assays and affinity purification of the U1 snRNP

RNA spanning the U1-70K exon 7a region (starting 46 nt upstream of the alternative 3′ splice site until 53 nt downstream of the third cryptic 5′ splice site) was *in vitro* transcribed (T7 High Yield RNA Synthesis Kit, NEB) from a PCR-generated DNA template. Purified transcripts (184 nt) were chemically 3′-biotinylated [41], and 60 pmol were incubated with 50 µl HeLa nuclear extract (CILBIOTECH, Mons, Belgium) in a total volume of 400 µl binding buffer (20 mM HEPES/KOH pH 7.5, 100 mM KCl, 10 mM MgCl_2_, 0.01% NP-40, 1 mM DTT) for 1 hour at room temperature. Bound material was pulled down via NeutrAvidin agarose beads (Thermo Scientific) for 2 hours at 4°C, and after several washing steps (20 mM HEPES/KOH pH 7.5, 200 mM KCl, 10 mM MgCl_2_, 0.01% NP-40, 1 mM DTT) bound proteins were analyzed by SDS-PAGE and Western blot, and bound U1 snRNA was detected by Northern blot hybridization.

The affinity purification of U1 snRNPs from HeLa nuclear extracts or whole cell extracts (after U1-70K knockdown) was according to Palfi *et al.* [42]; the experimental procedure is basically the same as described above for the *in vitro* binding assays but using a 3′-biotinylated 2′-*O*-methyl antisense RNA oligonucleotide (5′-GCCAGGUAAGUAU-3′) directed against the 5′ end of the U1 snRNA. Affinity-selected U1 snRNA was detected by Northern blotting, and co-purified proteins were examined by Western blot.

### *In vivo* rescue of U1C-knockout mutant zebrafish embryos by cRNA injection

ZfU1C-cRNA injection into *Danio rerio* embryos at the 1-cell stage was performed as described previously [22]. Phenotypically wildtype individuals were sorted into wildtype (wt), mutant (mut), and “rescued” (rsc) individuals. Single embryos were used to measure ZfU1C protein expression by Western blot, using specific antibodies against ZfU1C and γ-tubulin, as a control. Splicing patterns of zebrafish U1-70K were analyzed by RT-PCR using total RNA isolated from single embryos and specific primers against exons 7, 7a, and 8.

## Supporting Information

Figure S1 Stable U1C-deficient U1 snRNPs and RT-PCR validation of U1C-dependent alternative splicing changes in HeLa cells. (A) Affinity purification of U1 snRNP after U1C knockdown. After 72 hours of U1C knockdown in HeLa cells, whole-cell and nuclear extracts were prepared from control- (ctr) and U1C-knockdown (ΔC) cells. Nuclear extracts were used for affinity purification of the U1 snRNP, using a 2′-*O*-methyl-RNA antisense oligonucleotide. All extracts (lanes 1–4) and the purified material (lanes 5 and 6) were analyzed by SDS-PAGE and Western blot, detecting U1-70K, U1A, U1C, and, as a loading control, γ-tubulin. In addition, the U1 snRNA was detected by Northern blotting. (B) *In vitro* U1 snRNP binding to regulatory 5′ splice sites of U1-70K exon 7a after U1C knockdown. Nuclear extracts were prepared from both control- (ctr) and U1C-knockdown (ΔC) HeLa cells (10% input, lanes 1 and 3) and incubated with a biotinylated RNA containing the regulatory 5′ splice site sequences (as indicated in the schematic on the right). Bound material (bound, lanes 2 and 4) was pulled down via NeutrAvidin agarose, and proteins were analyzed by SDS-PAGE and Western blotting, using antibodies against U1-70K, U1A, and U1C; bound U1 snRNA was detected by Northern blot hybridization. (C–D) Alternative splicing of 28 U1C target genes (names above the lanes) was analyzed by RT-PCR, using total RNA from control- (ctr) and U1C-knockdown (ΔC) HeLa cells. Specific primers (indicated by the arrows in the schematics) were designed such that both alternative splice isoforms were amplified simultaneously. M, DNA size markers in bp. Asterisks mark unspecific PCR products, most likely primer dimers. (C) Increased exon skipping of 17 target genes after U1C knockdown. Top and lower bands represent exon inclusion and skipping products, respectively. In the case of *SNHG5*, the top band marked with an open circle is an unspecific PCR product. (D) U1C-dependent alternative 5′ splice site usage of 11 target genes: increased use of the proximal site. The top and lower bands reflect usage of the proximal and distal 5′ splice sites, respectively. In one case (*UFM1*), three alternative 5′ splice sites (labelled with 1, 2, and 3) are activated in the absence of U1C.
(PDF)

Figure S2 U1C-dependent alternative splicing of U1-70K is conserved between human, mouse, and zebrafish. (A) Conservation of U1-70K exon 7a region in human, mouse, and zebrafish. Sequences from 20 nt upstream of the alternative 3′ splice site until 11 nt downstream of regulatory 5′ splice site C from human (Hs; NM_003089), mouse (Mm; NM_009224), and zebrafish (Dr; NM_001003875) were aligned using ClustalW2. The positions of the alternative 3′ splice site (blue box), potential premature termination codons (red boxes with stop sign), and the three regulatory 5′ splice sites A, B, and C (green boxes) are highlighted; positions that are conserved in all three species are marked by asterisks below. (B) U1-70K alternative splicing after U1C knockdown in mouse myoblast cells. C2C12 cells were treated with an siRNA against U1C (ΔC), or as a control, with a luciferase-specific siRNA (ctr). 72 h after siRNA transfection, knockdown efficiencies were evaluated by Western blot analysis of whole cell lysates, detecting γ-tubulin (as a loading control) and U1C. Splicing patterns were analyzed by RT-PCR on total RNA, using specific primer sets (indicated in the schematic on the right) to detect exons 7-7a and 7–8 splicing, and, as a control, β-actin. Splicing products are depicted on the right of the gels. M, DNA size markers (in bp).
(PDF)

Figure S3 Analysis of cryptic 5′ splice site usage for U1-70K exon 7a inclusion. (A) Splicing of U1-70K after U1C or U1-70K knockdown. Alternative splicing of endogenous U1-70K mRNA after U1C knockdown was analyzed by RT-PCR on total RNA isolated from HeLa cells treated with an siRNA against U1C (ΔC), or a control siRNA (ctr), comparing untreated cells (−CHX, lanes 1 and 2) or cells after treatment with cycloheximide (+CHX, lanes 3 and 4). 72 hours after siRNA transfection, the U1-70K wildtype minigene construct was transfected into HeLa cells (ctr vs. Δ70K, lanes 5 and 6), and 24 hours later minigene splicing patterns were analyzed by RT-PCR. RT-PCR samples detecting exon 7a inclusion (primer from exon 7–8) from Figures 3B and 5C were analyzed on an *Agilent DNA 1000 Chip*. The identities of the splicing products are depicted on the right; the 5′ splice sites used for 7a inclusion are given below each lane, with capital letters marking the most frequently used splice site. (B) Splicing patterns of U1-70K minigenes (as indicated above the lanes) in control- (ctr) and U1C-knockdown (ΔC) HeLa cells. RT-PCR samples from Figure 4B detecting exon 7a inclusion and skipping (primers from 7–8) were analyzed on an *Agilent DNA 1000 Chip*. The identities of the splicing products are depicted on the right; the 5′ splice sites used for 7a inclusion are given below each lane, with capital letters marking the most frequently used splice site. Asterisks mark the three exon 7a inclusion bands in lane 1.
(PDF)

Table S1 Single-exon skipping targets upon U1C knockdown.
(PDF)

Table S2 Single-exon inclusion targets upon U1C knockdown.
(PDF)

Table S3 Proximal 5′ splice site usage upon U1C knockdown.
(PDF)

Table S4 Distal 5′ splice site usage upon U1C knockdown.
(PDF)

Table S5 List of primers used for validation RT-PCRs, U1-70K alternative splicing detection, and minigene cloning.
(PDF)
