# Supplementary figures and images for: A Novel Intra-U1 snRNP Cross-Regulation Mechanism: Alternative Splicing Switch Links U1C and U1-70K Expression
Source: PLoS Genet. 2013 Oct 17;9(10):e1003856. doi: 10.1371/journal.pgen.1003856 (PMC3798272; doi:10.1371/journal.pgen.1003856)

# Supplementary Figure S1

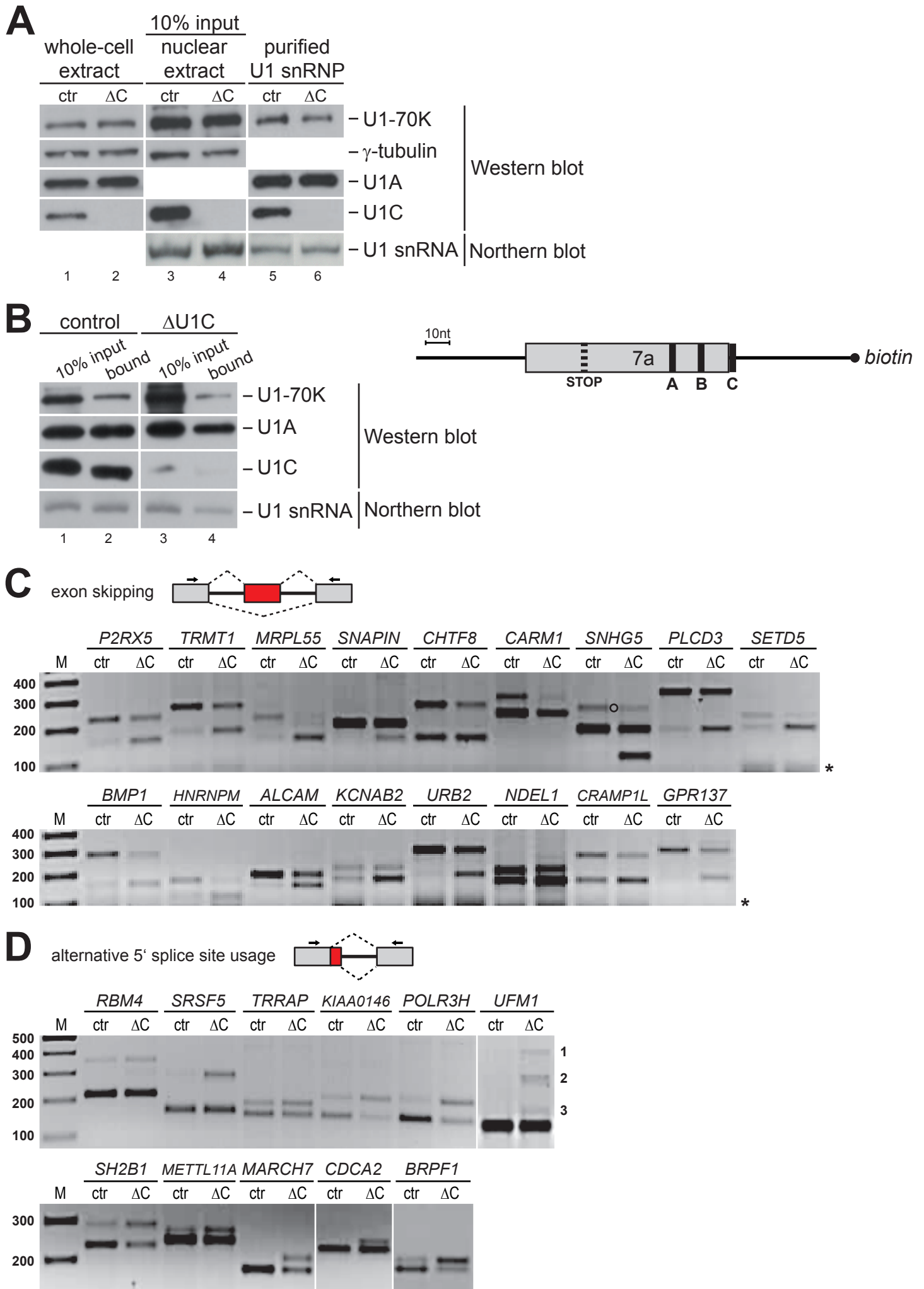

Supplement: Figure S1 — Stable U1C-deficient U1 snRNPs and RT-PCR validation of U1C-dependent alternative splicing changes in HeLa cells. (A) Affinity purification of U1 snRNP after U1C knockdown. After 72 hours of U1C knockdown in HeLa cells, whole-cell and nuclear extracts were prepared from control- (ctr) and U1C-knockdown (ΔC) cells. Nuclear extracts were used for affinity purification of the U1 snRNP, using a 2′-O-methyl-RNA antisense oligonucleotide. All extracts (lanes 1–4) and the purified material (lanes 5 and 6) were analyzed by SDS-PAGE and Western blot, detecting U1-70K, U1A, U1C, and, as a loading control, γ-tubulin. In addition, the U1 snRNA was detected by Northern blotting. (B) In vitro U1 snRNP binding to regulatory 5′ splice sites of U1-70K exon 7a after U1C knockdown. Nuclear extracts were prepared from both control- (ctr) and U1C-knockdown (ΔC) HeLa cells (10% input, lanes 1 and 3) and incubated with a biotinylated RNA containing the regulatory 5′ splice site sequences (as indicated in the schematic on the right). Bound material (bound, lanes 2 and 4) was pulled down via NeutrAvidin agarose, and proteins were analyzed by SDS-PAGE and Western blotting, using antibodies against U1-70K, U1A, and U1C; bound U1 snRNA was detected by Northern blot hybridization. (C–D) Alternative splicing of 28 U1C target genes (names above the lanes) was analyzed by RT-PCR, using total RNA from control- (ctr) and U1C-knockdown (ΔC) HeLa cells. Specific primers (indicated by the arrows in the schematics) were designed such that both alternative splice isoforms were amplified simultaneously. M, DNA size markers in bp. Asterisks mark unspecific PCR products, most likely primer dimers. (C) Increased exon skipping of 17 target genes after U1C knockdown. Top and lower bands represent exon inclusion and skipping products, respectively. In the case of SNHG5, the top band marked with an open circle is an unspecific PCR product. (D) U1C-dependent alternative 5′ splice site usage of 11 target genes: in [file pgen.1003856.s001.pdf]

# Supplementary Figure S2

**A**

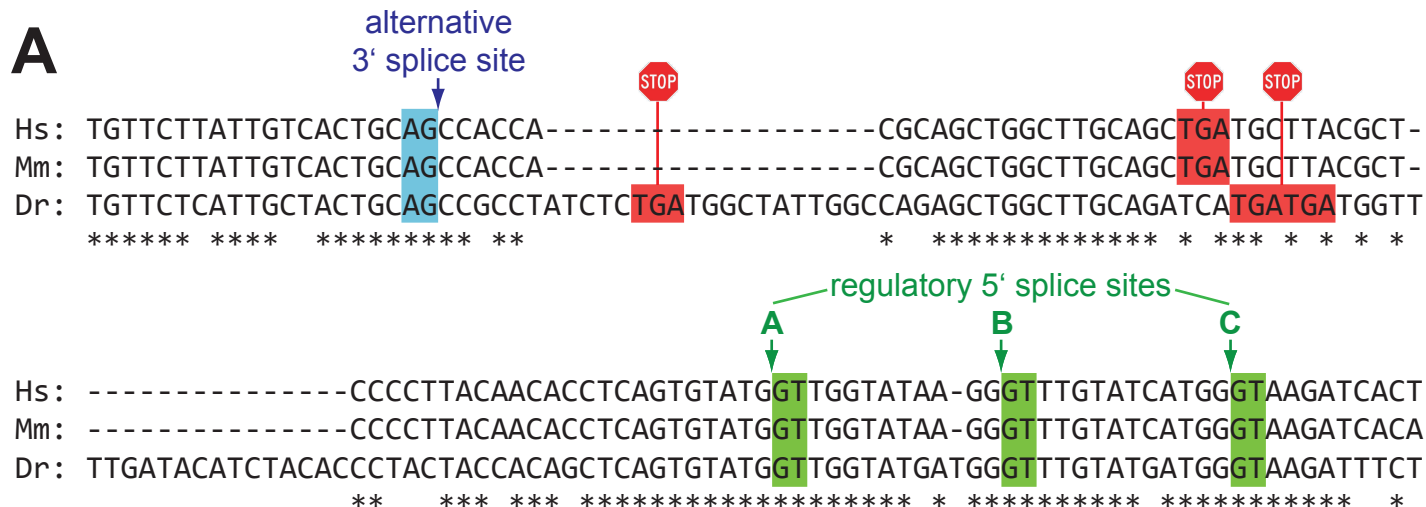

**B**

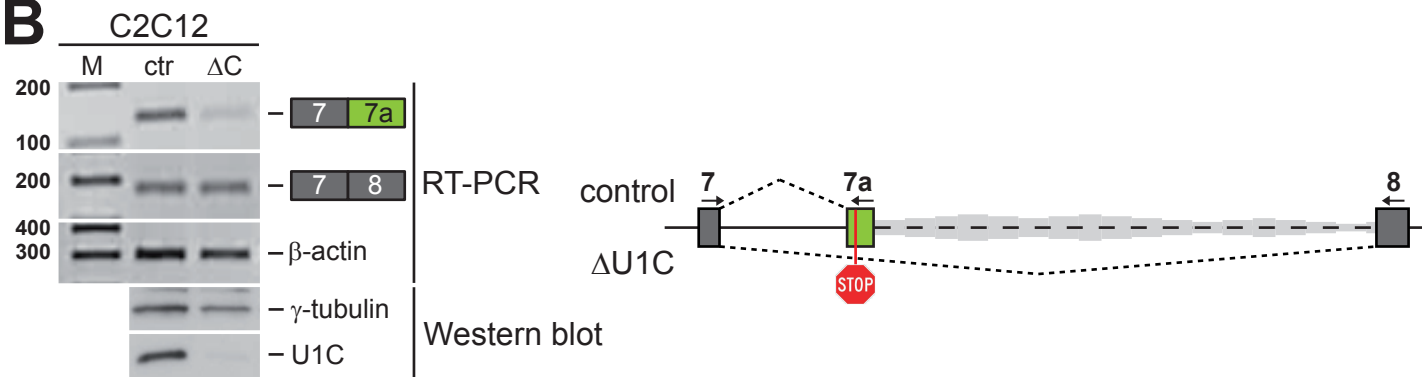

Supplement: Figure S2 — U1C-dependent alternative splicing of U1-70K is conserved between human, mouse, and zebrafish. (A) Conservation of U1-70K exon 7a region in human, mouse, and zebrafish. Sequences from 20 nt upstream of the alternative 3′ splice site until 11 nt downstream of regulatory 5′ splice site C from human (Hs; NM_003089), mouse (Mm; NM_009224), and zebrafish (Dr; NM_001003875) were aligned using ClustalW2. The positions of the alternative 3′ splice site (blue box), potential premature termination codons (red boxes with stop sign), and the three regulatory 5′ splice sites A, B, and C (green boxes) are highlighted; positions that are conserved in all three species are marked by asterisks below. (B) U1-70K alternative splicing after U1C knockdown in mouse myoblast cells. C2C12 cells were treated with an siRNA against U1C (ΔC), or as a control, with a luciferase-specific siRNA (ctr). 72 h after siRNA transfection, knockdown efficiencies were evaluated by Western blot analysis of whole cell lysates, detecting γ-tubulin (as a loading control) and U1C. Splicing patterns were analyzed by RT-PCR on total RNA, using specific primer sets (indicated in the schematic on the right) to detect exons 7-7a and 7–8 splicing, and, as a control, β-actin. Splicing products are depicted on the right of the gels. M, DNA size markers (in bp). (PDF) [file pgen.1003856.s002.pdf]

# Supplementary Figure S3

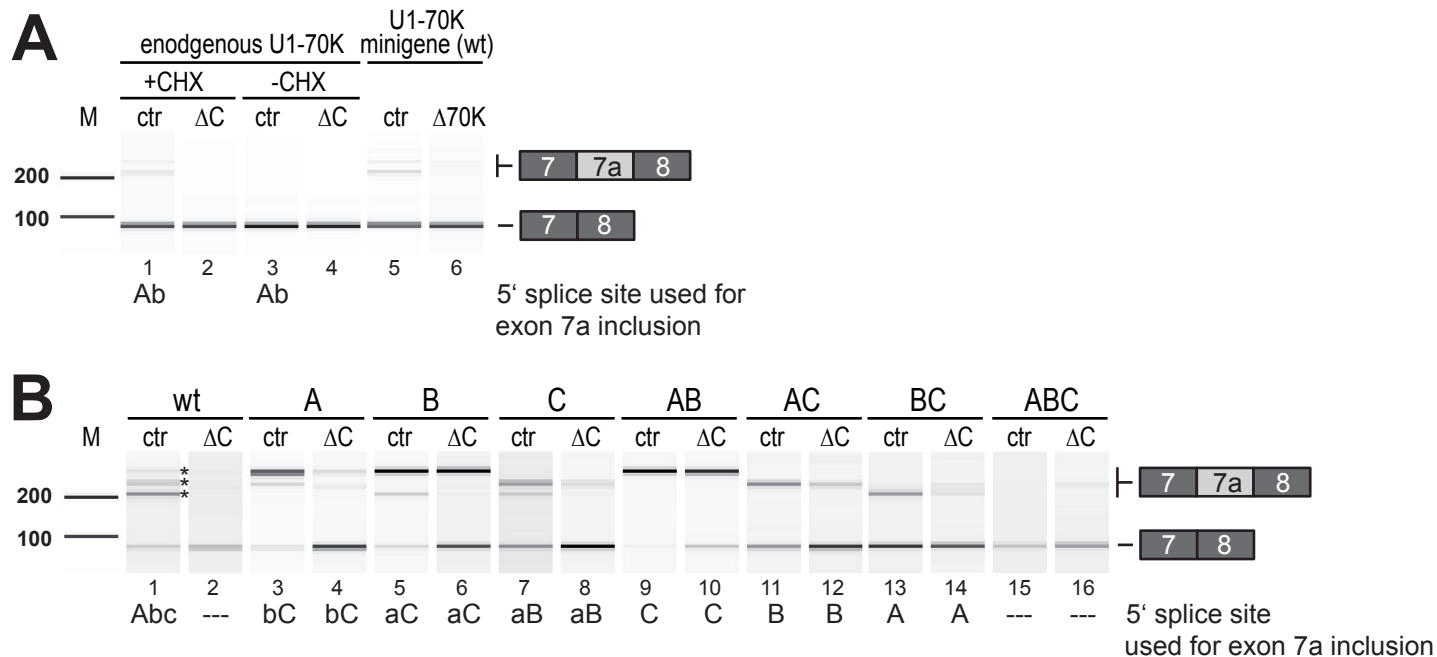

Supplement: Figure S3 — Analysis of cryptic 5′ splice site usage for U1-70K exon 7a inclusion. (A) Splicing of U1-70K after U1C or U1-70K knockdown. Alternative splicing of endogenous U1-70K mRNA after U1C knockdown was analyzed by RT-PCR on total RNA isolated from HeLa cells treated with an siRNA against U1C (ΔC), or a control siRNA (ctr), comparing untreated cells (−CHX, lanes 1 and 2) or cells after treatment with cycloheximide (+CHX, lanes 3 and 4). 72 hours after siRNA transfection, the U1-70K wildtype minigene construct was transfected into HeLa cells (ctr vs. Δ70K, lanes 5 and 6), and 24 hours later minigene splicing patterns were analyzed by RT-PCR. RT-PCR samples detecting exon 7a inclusion (primer from exon 7–8) from Figures 3B and 5C were analyzed on an Agilent DNA 1000 Chip. The identities of the splicing products are depicted on the right; the 5′ splice sites used for 7a inclusion are given below each lane, with capital letters marking the most frequently used splice site. (B) Splicing patterns of U1-70K minigenes (as indicated above the lanes) in control- (ctr) and U1C-knockdown (ΔC) HeLa cells. RT-PCR samples from Figure 4B detecting exon 7a inclusion and skipping (primers from 7–8) were analyzed on an Agilent DNA 1000 Chip. The identities of the splicing products are depicted on the right; the 5′ splice sites used for 7a inclusion are given below each lane, with capital letters marking the most frequently used splice site. Asterisks mark the three exon 7a inclusion bands in lane 1. (PDF) [file pgen.1003856.s003.pdf]
