# Supplementary material for: A Novel Intra-U1 snRNP Cross-Regulation Mechanism: Alternative Splicing Switch Links U1C and U1-70K Expression
Source: PLoS Genet. 2013 Oct 17;9(10):e1003856. doi: 10.1371/journal.pgen.1003856 (PMC3798272; doi:10.1371/journal.pgen.1003856)
Supplement: Table S1 — Single-exon skipping targets upon U1C knockdown. (PDF) [file pgen.1003856.s004.pdf]

Supplementary Table S1. Single-exon skipping targets upon U1C knockdown

gene_id	chromosome	strand	alternative_exon_position	skipping_junction_position
ADD1	chr4	+	2928369..2928402	2927839-2929898
AGPAT2	chr9	-	139571037..139571132	139569259-139571413
AKR1A1	chr1	+	46018108..46018235	46016827-46027461
ALCAM	chr3	+	105270988..105271026	105269103-105271312
ANXA2	chr15	-	60689457..60689537	60678285-60690142
ARMC6	chr19	+	19154801..19154883	19153686-19162431
ATG9A	chr2	-	220093156..220093207	220092775-220093732
AXL	chr19	+	41745599..41745625	41745219-41748788
BCAR1	chr16	-	75270780..75270896	75269884-75271081
BCL7B	chr7	-	72957878..72957974	72954382-72966497
BIN1	chr2	-	127808730..127808819	127808488-127816587
BMP1	chr8	+	22064367..22064494	22059441-22064816
C10orf137	chr10	+	127417572..127417673	127414407-127417927
C16orf13	chr16	-	685281..685340	684797-686094
C1orf43	chr1	-	154186369..154186422	154185100-154186933
C3orf17	chr3	-	112736295..112736449	112732879-112738389
C8orf59	chr8	-	86131465..86131592	86129731-86132535
C9orf114	chr9	-	131586026..131586173	131585112-131586351
CAMTA1	chr1	+	6880241..6880310	6845635-6885152
CARM1	chr19	+	11032051..11032119	11031803-11032291
CAST	chr5	+	96064857..96064913	96063234-96065316
CCDC99	chr5	+	169017760..169017833	169015579-169018052
CD46	chr1	+	207941124..207941168	207940540-207943666
CD97	chr19	+	14507154..14507285	14501891-14508470
CDA	chr1	+	20940335..20940392	20931532-20944945
CDC25C	chr5	-	137665242..137665336	137664218-137666676
CDIPT	chr16	-	29870763..29870844	29870655-29871902
CHD3	chr17	+	7810919..7811020	7810806-7811212
CHTF8	chr16	-	69154956..69155073	69154552-69155339
CKB	chr14	-	103986806..103986929	103986648-103987600
CLSTN1	chr1	-	9816539..9816568	9815367-9833330
CPNE7	chr16	+	89657535..89657668	89657466-89661775
CRAMP1L	chr16	+	1717336..1717401	1716601-1717963
CSNK1G3	chr5	+	122941033..122941056	122940524-122950036
DMWD	chr19	-	46287899..46287973	46287548-46288852
DNAJA3	chr16	+	4504812..4504928	4500498-4505547
DPH3	chr3	-	16305662..16305736	16302336-16306276
DROSHA	chr5	-	31515561..31515671	31515326-31521230

EIF4E2	chr2	+	233438973..233439051	233431924-233445614
EIF4G2	chr11	-	10823208..10823321	10822634-10823596
EIF4H	chr7	+	73604577..73604636	73604248-73609071
ERCC1	chr19	-	45917221..45917292	45917003-45918119
EWSR1	chr22	+	29678379..29678546	29674205-29682912
EXOC7	chr17	-	74087224..74087316	74085401-74090495
FAM107B	chr10	-	14595321..14595386	14572514-14646239
FAM122B	chrX	-	133923610..133923666	133923296-133927845
FBXO22	chr15	+	76196832..76196970	76196444-76205544
FHL2	chr2	-	106013104..106013154	106002997-106015299
FIS1	chr7	-	100887288..100887420	100884187-100888241
FKBP10	chr17	+	39973310..39973455	39969531-39974341
FLNA	chrX	-	153581922..153582095	153581825-153582283
FLNB	chr3	+	58117654..58117746	58116635-58118535
GGCT	chr7	-	30540152..30540297	30538554-30544185
GPR137	chr11	+	64056075..64056193	64055940-64056509
GPR56	chr16	+	57675503..57675620	57662714-57684165
GSTM1	chr1	+	110232893..110233186	110231947-110235828
GSTP1	chr11	+	67353575..67353682	67352712-67353860
GTF2I	chr7	+	74133198..74133260	74131270-74143124
GUSB	chr7	-	65435269..65435353	65432894-65439282
HCFC1R1	chr16	-	3073475..3073531	3073362-3073848
HNRNPA1	chr12	+	54676863..54677018	54676658-54677596
HNRNPA2B1	chr7	-	26237451..26237486	26237352-26240192
HNRNPAB	chr5	+	177637133..177637273	177636448-177637554
HNRNPH1	chr5	-	179042547..179042596	179041960-179043127
HTRA2	chr2	+	74759746..74759841	74759052-74759947
IL17RC	chr3	+	9970264..9970314	9970170-9971536
IL17RC	chr3	+	9974504..9974542	9974387-9974637
INCENP	chr11	+	61908972..61908983	61908516-61912454
ISOC2	chr19	-	55967003..55967212	55966697-55967716
ITM2C	chr2	+	231738132..231738272	231729860-231740335
KCNAB2	chr1	+	6101891..6101932	6100705-6132815
LAMTOR1	chr11	-	71809335..71809461	71808960-71809827
LDHA	chr11	+	18426996..18427119	18425358-18428664
MADD	chr11	+	47348289..47348358	47346128-47350209
MAPK9	chr5	-	179713975..179714067	179707608-179718848
MAPKAP1	chr9	-	128321912..128322088	128305447-128347834
MFAP5	chr12	-	8804258..8804287	8803185-8807033
MFF	chr2	+	228193394..228193505	228190143-228195342

MFF	chr2	+	228211942..228212100	228205096-228220393
MINK1	chr17	+	4795697..4795807	4795529-4795951
MKL1	chr22	-	40990678..40990739	40948371-41032482
MKNK1	chr1	-	47025906..47025949	47024472-47027150
MLX	chr17	+	40720488..40720577	40719673-40720855
MPRIP	chr17	+	17083921..17083983	17083402-17088137
MRPL55	chr1	-	228296656..228296722	228296019-228296850
MRPS11	chr15	+	89015858..89015956	89011255-89018341
MRPS18C	chr4	+	84379499..84379582	84378111-84380893
MTDH	chr8	+	98711982..98712080	98703416-98718854
MYL6	chr12	+	56554410..56554454	56554104-56555171
MYL9	chr20	+	35176435..35176596	35173471-35177480
NAP1L4	chr11	-	2970457..2970494	2966876-2972489
NCOR2	chr12	-	124858959..124859009	124857156-124862783
NDEL1	chr17	+	8366638..8366672	8363478-8370248
NF2	chr22	+	30079009..30079053	30077590-30090741
NFE2L1	chr17	+	46134394..46134483	46133960-46134706
NFIC	chr19	+	3456548..3456633	3452664-3462750
NIPA2	chr15	-	23033278..23033413	23021429-23033894
NPM1	chr5	+	170827843..170827929	170827214-170832306
NPNT	chr4	+	106833345..106833395	106819158-106848493
NPRL3	chr16	-	167300..167374	162774-169125
NRM	chr6	-	30657053..30657229	30656719-30657824
P2RX5	chr17	-	3594250..3594321	3593974-3594938
PABPC4	chr1	-	40029508..40029594	40029413-40030143
PCBP2	chr12	+	53858544..53858636	53856351-53859716
PCBP2	chr12	+	53861589..53861627	53861077-53862561
PICALM	chr11	-	85701293..85701442	85695016-85707869
PLCD3	chr17	-	43190231..43190380	43190072-43190490
POC1A	chr3	-	52130585..52130728	52110001-52156395
POP5	chr12	-	121017577..121017726	121017400-121018918
PPHLN1	chr12	+	42745687..42745851	42729776-42748963
PPP2R4	chr9	+	131890243..131890347	131885417-131891264
PQLC1	chr18	-	77693969..77694022	77679400-77703329
PRC1	chr15	-	91512309..91512350	91510432-91512677
PRPF38B	chr1	+	109236212..109236264	109235489-109238324
PTBP1	chr19	+	805492..805569	805187-806408
PTPMT1	chr11	+	47591252..47591443	47587538-47593023
PUF60	chr8	-	144906483..144906569	144904083-144911450
PXDN	chr2	-	1691404..1691475	1687923-1695700

RAD1	chr5	-	34913575..34913683	34911917-34914800
RAF1	chr3	-	12647700..12647798	12645788-12650265
RBM39	chr20	-	34317384..34317449	34317287-34319863
RHOC	chr1	-	113247722..113247790	113246428-113249700
RNF212	chr4	-	1102131..1102192	1090627-1107144
ROBO1	chr3	-	78696779..78696805	78695340-78700882
RPL21	chr13	+	27828357..27828418	27827980-27829379
RPN2	chr20	+	35860699..35860794	35858462-35862423
RPS24	chr10	+	79799962..79799983	79797062-79800373
RWDD1	chr6	+	116895221..116895334	116892818-116901458
S100A4	chr1	-	153517434..153517994	153517285-153518229
SBF1	chr22	-	50895463..50895540	50895102-50897684
SDCCAG3	chr9	-	139303451..139303519	139302390-139304542
SDHA	chr5	+	251107..251583	240591-254508
SEC16A	chr9	-	139340097..139340171	139338352-139341307
SETD5	chr3	+	9476274..9476314	9476169-9476508
SFXN4	chr10	-	120920567..120920593	120920481-120921852
SHARPIN	chr8	-	145153984..145154108	145153897-145154180
SHMT1	chr17	-	18238873..18238989	18236602-18243357
SIKE1	chr1	-	115322731..115322836	115321905-115323070
SKA2	chr17	-	57196680..57196856	57189706-57208642
SLC3A2	chr11	+	62639049..62639141	62623853-62648491
SNAPIN	chr1	+	153631614..153631660	153631362-153631924
SNHG5	chr6	-	86387513..86387593	86387210-86387672
SRRM1	chr1	+	24989674..24989715	24989295-24993306
SRRT	chr7	+	100478317..100478390	100473333-100478906
SSFA2	chr2	+	182785324..182785389	182784173-182786675
SUN2	chr22	-	39150647..39150711	39148670-39151768
THOC5	chr22	-	29946717..29946832	29945147-29949437
TMEM107	chr17	-	8077838..8079344	8077590-8079518
TPD52L2	chr20	+	62507169..62507228	62505169-62514072
TRMT1	chr19	-	13220729..13220815	13220652-13220972
TRPT1	chr11	-	63993258..63993341	63993052-63993522
TSPAN17	chr5	+	176078617..176078901	176074703-176079744
TSPAN4	chr11	+	865513..865614	864511-865694
TUFM	chr16	-	28856635..28856801	28856376-28857233
TXNRD1	chr12	+	104682709..104682818	104681124-104705068
UBTF	chr17	-	42289712..42289822	42289374-42290187
URB2	chr1	+	229781606..229781716	229779440-229783257
USP37	chr2	-	219344373..219344438	219341704-219346793

WDR45	chrX	-	48935302..48935406	48934409-48935496
WDR90	chr16	+	710058..710161	709376-710612
WNK1	chr12	+	980431..980514	971436-987378
YAP1	chr11	+	102080248..102080295	102076805-102094353
YIPF1	chr1	-	54320674..54320724	54317943-54325729
YKT6	chr7	+	44250622..44250723	44247797-44251846
ZMIZ2	chr7	+	44799750..44799827	44799059-44800024
ZNF207	chr17	+	30688487..30688534	30687986-30689933
ZNF207	chr17	+	30693684..30693776	30692506-30694791
ZNF384	chr12	-	6781516..6781698	6780004-6782382
ZRANB2	chr1	-	71531361..71531435	71530820-71532459
