# Supplementary material for: A Novel Intra-U1 snRNP Cross-Regulation Mechanism: Alternative Splicing Switch Links U1C and U1-70K Expression
Source: PLoS Genet. 2013 Oct 17;9(10):e1003856. doi: 10.1371/journal.pgen.1003856 (PMC3798272; doi:10.1371/journal.pgen.1003856)
Supplement: Table S2 — Single-exon inclusion targets upon U1C knockdown. (PDF) [file pgen.1003856.s005.pdf]

Supplementary Table S2. Single-exon inclusion targets upon U1C knockdown

gene_id	chromosome	strand	alternative_exon_position	skipping_junction_position
ABLIM1	chr10	-	116213138..116213242	116211430-116225457
ANAPC5	chr12	-	121747773..121747814	121747666-121756080
ASNS	chr7	-	97501031..97501308	97499125-97501663
C16orf61	chr16	-	81034853..81034939	81031034-81040339
C20orf3	chr20	-	24945011..24945034	24944658-24949528
CALM2	chr2	-	47399553..47399598	47397903-47403580
CCNL2	chr1	-	1323872..1324123	1323445-1325610
CLTC	chr17	+	57764362..57764382	57763169-57767997
DROSHA	chr5	-	31467249..31467273	31466388-31468046
EZH2	chr7	-	148516070..148516151	148515209-148516688
FAM13B	chr5	-	137292166..137292231	137290065-137295305
FLNB	chr3	+	58127585..58127656	58124256-58128377
FNBP4	chr11	-	47747289..47747388	47746330-47752926
GOPC	chr6	-	117898611..117898634	117896515-117900063
GTF3C5	chr9	+	135930331..135930351	135930196-135931399
HERC4	chr10	-	69718870..69718893	69716733-69726440
HNRNPH1	chr5	-	179048844..179048977	179048398-179050038
HNRPDL	chr4	-	83346716..83346820	83346036-83347190
IVNS1ABP	chr1	-	185275617..185275757	185274775-185276146
KIF21A	chr12	-	39724044..39724064	39720126-39724548
NFYC	chr1	+	41232590..41232646	41232375-41235028
ORMDL1	chr2	-	190647740..190647849	190647328-190648995
PRMT1	chr19	+	50183129..50183182	50180573-50183744
RBM3	chrX	+	48434203..48434471	48434055-48434702
RBM39	chr20	-	34301216..34301779	34301018-34302107
RBMS1	chr2	-	161138768..161138815	161137875-161141286
SLC25A38	chr3	+	39430853..39430880	39425284-39430986
SNX14	chr6	-	86248556..86248582	86246642-86251703
SPTAN1	chr9	+	131371930..131371944	131371563-131373993
SRRM1	chr1	+	24973570..24973699	24973280-24975350
TEAD1	chr11	+	12900436..12900447	12886447-12901255
TMED5	chr1	-	93621593..93621641	93620445-93621857
TNRC6A	chr16	+	24804794..24804970	24803138-24805865
TRIM16L	chr17	+	18627083..18627144	18625645-18630838
UBE2B	chr5	+	133724668..133724743	133724104-133725916
UBE2I	chr16	+	1362348..1362604	1359774-1364021
WNK1	chr12	+	988739..989197	987527-989887
