# Supplementary material for: A Novel Intra-U1 snRNP Cross-Regulation Mechanism: Alternative Splicing Switch Links U1C and U1-70K Expression
Source: PLoS Genet. 2013 Oct 17;9(10):e1003856. doi: 10.1371/journal.pgen.1003856 (PMC3798272; doi:10.1371/journal.pgen.1003856)
Supplement: Table S3 — Proximal 5′ splice site usage upon U1C knockdown. (PDF) [file pgen.1003856.s006.pdf]

Supplementary Table S3. Proximal 5' splice site usage upon U1C knockdown				
**gene_id**	**chromosome**	**strand**	**distal_5'SS_junction_position**	**proximal_5'SS_junction_position**
ALDOA	chr16	+	30080299-30080627	30080311-30080627
ALG13	chrX	+	110924527-110925360	110924652-110925360
ARIH2	chr3	+	49008137-49011132	49008274-49011132
ARMCX3	chrX	+	100878266-100878776	100878420-100878776
ASCC1	chr10	-	73973089-73975965	73973089-73975540
AURKA	chr20	-	54965721-54967210	54965721-54966999
BAG1	chr9	-	33262828-33264222	33262828-33264204
BRPF1	chr3	+	9783837-9784628	9783855-9784628
BUD31	chr7	+	99006627-99007657	99006868-99007657
BZW2	chr7	+	16685913-16705062	16685929-16705062
C7orf49	chr7	-	134853812-134855404	134853812-134855151
CCNK	chr14	+	99947816-99958963	99947951-99958963
CDKN1A	chr6	+	36646575-36651874	36646716-36651874
CENPW	chr6	+	126661545-126667351	126661590-126667351
CKS1B	chr1	+	154947280-154950463	154947388-154950463
CNOT1	chr16	-	58568299-58570893	58568299-58570872
COX7A2	chr6	-	75950101-75950892	75950101-75950839
CRK	chr17	-	1326944-1340084	1326944-1339914
DDX56	chr7	-	44609502-44609614	44609502-44609583
DLG4	chr17	-	7107571-7111493	7107571-7111394
DLGAP4	chr20	+	35154409-35155216	35154480-35155216
DNAJC11	chr1	-	6697564-6698355	6697564-6698143
DNMT1	chr19	-	10246963-10247761	10246963-10247752
DYRK1A	chr21	+	38878526-38884214	38878657-38884214
EHMT2	chr6	-	31852576-31852689	31852576-31852668
EIF4B	chr12	+	53428493-53431193	53428508-53431193
EPHX1	chr1	+	225997871-226016426	225998317-226016426
EXOC3	chr5	+	462422-464405	462644-464405
EXOSC2	chr9	+	133570980-133572969	133570993-133572969
FAM214A	chr15	-	52877141-52879348	52877141-52879253
FAM82A2	chr15	-	41046988-41047397	41046988-41047281
FIBP	chr11	-	65652657-65653001	65652657-65652980
FPGS	chr9	+	130569967-130570513	130570054-130570513
GUSB	chr7	-	65441189-65444386	65441189-65444373
HDLBP	chr2	-	242169376-242169568	242169376-242169484
HMGB2	chr4	-	174254820-174255423	174254820-174255397
HNRNPC	chr14	-	21698525-21699156	21698525-21699117
HNRNPH1	chr5	-	179050165-179050596	179050165-179050282

HNRNPU	chr1	-	245026032-245026976	245026032-245026919
HSD17B10	chrX	-	53458542-53458773	53458542-53458746
ITPR1	chr3	+	4716905-4718298	4716932-4718298
KRT7	chr12	+	52631353-52632464	52631501-52632464
LAMA4	chr6	-	112575493-112575661	112575493-112575644
LEPRE1	chr1	-	43212523-43212943	43212523-43212924
MAD2L2	chr1	-	11736197-11736905	11736197-11736866
MARCH7	chr2	+	160615846-160619391	160615869-160619391
MCM4	chr8	+	48873774-48874076	48873986-48874076
MEST	chr7	+	130126236-130135209	130126360-130135209
METTL11A	chr9	+	132388574-132394929	132388600-132394929
MOCS2	chr5	-	52404473-52405542	52404473-52405081
MRPL21	chr11	-	68660461-68660871	68660461-68660790
MRPL36	chr5	-	1799061-1799906	1799061-1799885
MRPS14	chr1	-	174987712-174992500	174987712-174992408
MTCH1	chr6	-	36938470-36940479	36938470-36940428
NNMT	chr11	+	114168880-114182767	114168916-114182767
NUMA1	chr11	-	71780957-71791504	71780957-71791366
NUP155	chr5	-	37364486-37370923	37364486-37370768
OS9	chr12	+	58112159-58112776	58112204-58112776
OSGEP	chr14	-	20916970-20917123	20916970-20917061
PABPC4	chr1	-	40035674-40036906	40035674-40036769
PARK7	chr1	+	8021795-8022823	8021853-8022823
PCID2	chr13	-	113854830-113862913	113854830-113862338
PLEKHB2	chr2	+	131890564-131897740	131890588-131897740
PLK1	chr16	+	23700997-23701181	23701093-23701181
PLK1	chr16	+	23690661-23691405	23690821-23691405
POLR3H	chr22	-	41928161-41928663	41928161-41928608
PPP1R8	chr1	+	28157402-28159267	28157877-28159267
PQBP1	chrX	+	48755365-48755775	48755376-48755775
PRPF40A	chr2	-	153572640-153573870	153572640-153573744
PRRC2C	chr1	+	171553665-171556142	171553680-171556142
PSMA7	chr20	-	60714253-60714837	60714253-60714752
RBM4	chr11	+	66406223-66407171	66406364-66407171
RHOC	chr1	-	113246428-113247722	113246428-113247675
RMND5B	chr5	+	177565259-177569584	177565397-177569584
RNF31	chr14	+	24617622-24617851	24617686-24617851
RPL13	chr16	+	89628799-89629292	89628853-89629292
RPL15	chr3	+	23958730-23959341	23958786-23959341
RPL34	chr4	+	109541756-109543107	109541868-109543107

RPL35A	chr3	+	197678182-197680874	197678199-197680874
RPS20	chr8	-	56986718-56986940	56986718-56986813
RPS6	chr9	-	19379616-19380188	19379616-19380086
RPS9	chr19	+	54704756-54705028	54704829-54705028
RTEL1	chr20	+	62326833-62327131	62327003-62327131
SDCCAG3	chr9	-	139300430-139301597	139300430-139301398
SEC31A	chr4	-	83803093-83812241	83803093-83811904
SH2B1	chr16	+	28884026-28884491	28884079-28884491
SMARCB1	chr22	+	24143268-24145482	24143322-24145482
SMG7	chr1	+	183511499-183513488	183511637-183513488
SNF8	chr17	-	47010708-47013532	47010708-47013493
SRSF5	chr14	+	70233972-70234855	70234097-70234855
STT3B	chr3	+	31674639-31677476	31674676-31677476
STX16	chr20	+	57227143-57234679	57227194-57234679
TATDN1	chr8	-	125528271-125531059	125528271-125530983
TAZ	chrX	+	153641589-153641819	153641695-153641819
TCEAL4	chrX	+	102840552-102841147	102840611-102841147
TFRC	chr3	-	195803993-195808914	195803993-195808702
THAP4	chr2	-	242524140-242541311	242524140-242541230
TMED10	chr14	-	75601709-75602463	75601709-75602366
TMEM106C	chr12	+	48359955-48360466	48360012-48360466
TMEM55B	chr14	-	20929004-20929343	20929004-20929322
TMEM93	chr17	+	3572193-3572392	3572306-3572392
TRAF2	chr9	+	139794972-139802522	139795128-139802522
TRRAP	chr7	+	98581082-98581716	98581115-98581716
UBAP2L	chr1	+	154207235-154207672	154207268-154207672
UFM1	chr13	+	38924192-38928376	38924364-38928376
UNC45A	chr15	+	91478560-91478774	91478605-91478774
UQCR10	chr22	+	30163537-30165667	30163596-30165667
USP5	chr12	+	6972472-6972994	6972541-6972994
VARS	chr6	-	31749729-31749864	31749729-31749818
WDR26	chr1	-	224612356-224619227	224612356-224619179
WDR4	chr21	-	44296877-44299517	44296877-44299197
