# Supplementary material for: A Novel Intra-U1 snRNP Cross-Regulation Mechanism: Alternative Splicing Switch Links U1C and U1-70K Expression
Source: PLoS Genet. 2013 Oct 17;9(10):e1003856. doi: 10.1371/journal.pgen.1003856 (PMC3798272; doi:10.1371/journal.pgen.1003856)
Supplement: Table S4 — Distal 5′ splice site usage upon U1C knockdown. (PDF) [file pgen.1003856.s007.pdf]

Supplementary Table S4. Distal 5' splice site usage upon U1C knockdown

gene_id	chromosome	strand	distal_5'SS_junction_position	proximal_5'SS_junction_position
ANKRD13B	chr17	+	27935017-27935213	27935128-27935213
C11orf24	chr11	-	68035719-68039325	68035719-68039114
C12orf57	chr12	+	7053728-7054934	7053815-7054934
CHD3	chr17	+	7812656-7813746	7812702-7813746
GSN	chr9	+	124088960-124089586	124089070-124089586
HMGA1	chr6	+	34208659-34210489	34208692-34210489
NOP56	chr20	+	2636680-2637047	2636860-2637047
RPL14	chr3	+	40498965-40499381	40499238-40499381
RPL7	chr8	-	74205032-74206101	74205032-74205834
SRSF9	chr12	-	120901925-120903559	120901925-120903430
TUSC2	chr3	-	50363617-50363816	50363617-50363788
UBE2D3	chr4	-	103747793-103748749	103747793-103748584
