# Supplementary material for: A Novel Intra-U1 snRNP Cross-Regulation Mechanism: Alternative Splicing Switch Links U1C and U1-70K Expression
Source: PLoS Genet. 2013 Oct 17;9(10):e1003856. doi: 10.1371/journal.pgen.1003856 (PMC3798272; doi:10.1371/journal.pgen.1003856)
Supplement: Table S5 — List of primers used for validation RT-PCRs, U1-70K alternative splicing detection, and minigene cloning. (PDF) [file pgen.1003856.s008.pdf]

Supplementary Table S5. List of primers used for validation RT-PCRs, U1-70K alternative splicing detection, and minigene cloning

The primer names provide information on the target gene as well as on the location and direction (forward or reverse).

application	primer	sequence
U1C target validation	BMP1 E16 fwd	ACAAGGACGAGTGCTCCAAGG
	BMP1 E18 rev	TGGGACTCGATGTCCATCTCC
	HNRNPM E12 fwd	GGCCCTTTGGTGGTGGTATGG
	HNRNPM E14 rev	CAGGACCCATCCTCTCGATCC
	ALCAM E12 fwd	GCACAGCAGAAAACCAACTGGAG
	ALCAM E14 rev	AGCCAGTAGACGACACCAGCAAC
	KCNAB2 E2 fwd	CCGAGCAAGTCTGGATAAGTGAGG
	KCNAB2 E5 rev	CCTCCGAAGGTCACCCATGTTC
	URB2 E5 fwd	GAGCCTCATTTGGGAGCCTTG
	URB2 E7 rev	CATCTAAGACAGGCCCGACCAC
	NDEL1 E8 fwd	GAATTTTGCAAAGGACCAAGCATCACG
	NDEL1 E10 rev	AGCCCAGACCAGGAGGAGGAG
	CRAMP1L E13 fwd	CCAGTCCCACAACGTTTGTTCC
	CRAMP1L E15 rev	CACCCGTCACCTCGTCTGAAG
	GPR137 exon 5 fwd	TCCTCTTCGTGTGGGAGCTACTG
	GPR137 exon 7 rev	GGGAGAAGAGCAGAGGAGTGGTC
	P2RX5 exon 2 fwd	CTCCCTGCAGAGTGCTGTCATC
	P2RX5 exon 4 rev	CAGTCGCTGTCCTTGGAGCAC
	TRMT1 exon 7 fwd	CCTGAGAATCGTCCTGCACAGC
	TRMT1 exon 9 rev	GACCACAGGCTGCAGAGAACTTG
	SETD5 exon 5 fwd	ATCCCTCGTTCTGACCTGAATGGC
	SETD5 exon 6 rev	CTTCCGCCGATGAAGTCTAATAACC
	MRPL55 exon 1 fwd	GCCTCGTCCCTGGTCTTAGTTCC
	MRPL55 exon 3 rev	CATTCCTCCTTACAGCCCCAGTG
	SNAPIN exon 1 fwd	CCGAAGGGCTGCTGGAGTTC
	SNAPIN exon 3 rev	GCGTCGCCGGGCATTAAGTAGC
	CHTF8 exon 2 fwd	GACAGAAAGCTCATGGTGCAAATTG
	CHTF8 exon 4 rev	CAAGCTCATCACAGTCCTGATCC
	PLCD3 exon 13 fwd	CCGAGAAGCCACACTCCATTGTG
	PLCD3 exon 15 rev	CTGGATGCGGATTTGGATGAAGAGC
	CARM1 exon 14 fwd	AGTGTTATTGCCAGTGGCTCCAGC
	CARM1 exon 16 rev	CATCATTTGGTTTCCTGGTGCTGTC
	SNHG5 exon 3 fwd	GGAAAATCGCTTGGTTAAAACCTGACAC
	SNHG5 exon 5 rev	AGAAATCGTTGCAACTTTGGAACTTCC
	UFM1 E2 fwd	GGTTTCCTTTAAGATCACGCTGACG
	UFM1 E3 rev	CTGCTGCAAACTTTAAGACTGCTGTG
	RBM4 E1 fwd	GAGGCCCTGCTGGTTTCTGTG
	RBM4 E2 rev	GTAATGGTGCAGGTTGCGTATGGC
	SH2B1 E8 fwd	CAGCACCTGTGGTTCCAGTCC
	SH2B1 E9 rev	CACAGTCACTCGCTCCGAAGG
	METTL11A E1 fwd	CCTCCCTTCCTCTTCCCCATC
	METTL11A E2 rev	GGGAGCTGTTGATGTCGATGC
	MARCH7 E7 fwd	TTGGAGCTTAACCTGGAGGATTTTG
	MARCH7 E8 rev	CGGACACGTGTGCTTGGTTC
	CDCA2 E14 fwd	ATTCCCGAGCTGCCTGAAGTC
	CDCA2 E15 rev	GCATTGCCAAGCGATGAGGAAC
	TCF25 E11 fwd	CATGCTGCTGCTCATCGACCAC
	TCF25 E12 rev	TGCTCACACTCAGGGAGGTCTG

	SRSF5 exon 1 fwd	AGTTGTGGAGTGGCGTAGACGAG
	SRSF5 exon 2 rev	GTCTCCCGATGAATACCCGACAG
	BRPF1 exon 6 fwd	TGCAGCTGACTCCTTTCCTCATC
	BRPF1 exon 7 rev	GCCTCCAAGTTCTGCTTCATGGTG
	TRRAP exon 58 fwd	ATGAAGACGGTGGTGAAGACCTGG
	TRRAP exon 59 rev	TGAACTGGGATCATGCTGAGAGC
	KIAA0156 exon 18 fwd	CGCCTTTTCCTGTGGGGACTG
	KIAA0156 exon 19 rev	CAAACCTCAGCAGGGAGGAAATGC
	POLR3H exon 4 fwd	CATTTTCGCTGCGTGGTGTTTCATCC
	POLR3H exon 5 rev	AACTTGGCTGGCTGCTGCAGTG
**U1-70K alternative splicing**	U1-70K exon 7 fwd	CATGGTCTACAGTAAGCGGTCAGGAAAGC
	U1-70K exon 7a rev (suitable for human and mouse)	GAGGTGTTGTAAGGGGAGCGTAAG
	U1-70K exon 8 rev	CAAGGACCCTCCTGCCATCAATC
	mU1-70K exon 7 fwd	CACATGGTATACAGTAAACGCTCTGG
	mU1-70K exon 8 rev	CCTCCAGCCCTTCACAGTCC
	zfU1-70K exon 7 fwd	AACTGGAAAACCAAGAGGCTATGCG
	zfU1-70K exon 7a rev	GAGCTGTGGTAGTAGGGTGTAGATG
	zfU1-70K exon 8 rev	GGCGCCATCCTTTTACCGTTCG
	β-actin exon 4 fwd (suitable for human and mouse)	TGGACTTCGAGCAAGAGATG
	β-actin exon 5 rev (suitable for human and mouse)	GTGATCTCCTTCTGCATCCTG
	zf β-actin exon 1fwd	GATCTTCACTCCCCTTGTTCA
	zf β-actin exon 2 rev	ATGTCTGGGTCGTCCAACAA
**U1C & U1-70K mRNA detection**	U1C exon 1fwd	GAATTCCAGAGCAACATGCCC
	U1C exon 3 fwd	CAGAAATGGATGGAAGAGCAG
	U1C exon 4 rev	CAAGGAAAGATACCTCCTACTCC
	U1-70K exon 2 fwd	TTGGCAAGATGACCCAGTTCCTG
	U1-70K exon 3 rev	GCTCCTCTCGGGTTTCAGCAC
**U1-70K minigene cloning**	U1-70K exon 7 BamHI fwd	CTAGTCGGATCCATACACATGGTCTACAGTAAGCGG
	U1-70K intron 7 downstream cut fwd 1	GGCCCTTTCCTCCCTGTAGCTTTAGCTGA
	U1-70K intron 7 downstream cut rev	GAATCCCCGCCACCCAGCCCTGGCCTAGG
	U1-70K intron 7 dowmstream cut fwd 2	GGCTGGGTGGCGGGGATTCTGTAGAGCTG
	U1-70K exon 8 EcoRI rev	GACTAGGAATTCCTAGCCGCCGGGGCCT
	U1-70K intron 6 fwd	TGAAGGACAGAGAGGGTCTGAGG
	U1-70K exon 7 rev	AGTGCATGTCTCGCTCGTGTTC
	U1-70K exon 7 fwd 1	GAACACGAGCGAGACATGCACT
	U1-70K intron 7 rev 1	CTCCCCAGGTTCACACACACAC
	U1-70K exon 7 fwd 2	GTGTGTGTGTGAACCTGGGGAG
	U1-70K intron 7 rev 2	GGAAGAGAGGGCAGGTTCAAAGG
	U1-70K exon 7 fwd 3	CCTTTGAACCTGCCCTCTCTTCC
	U1-70K intron 7 rev 3	ATTAATACGGCGGCCCCTGGAC
	U1-70K exon 7 fwd 4	GTCCAGGGGCCGCCGTATTAAT
	U1-70K intron 7 rev 4	CTCCTGAGCTGCCTGGCTAAG
	U1-70K exon 7 fwd 5	CTTAGCCAGGCAGCTCAGGAG

	U1-70K intron 7 rev 5	GTCATCAGAGGCTGGGTGGAAC
**U1-70K minigene mutagenesis**	U1-70K 5’ss A mut fwd	CACCTCAGTGTATGACTGGTATAAGG
	U1-70K 5’ss A mut rev	CCTTATACCAGTCATACACTGAGGTG
	U1-70K 5’ss B mut fwd	TAAGGACTTGTATCATGGGTAAGATCAC
	U1-70K 5’ss B mut rev	GTGATCTTACCCATGATACAAGTCCTTA
	U1-70K 5’ss C mut fwd	TAAGGGTTTGTATCATGGACAAGATCAC
	U1-70K 5’ss C mut rev	GTGATCTTGTCCATGATACAAACCCTTA
	U1-70K 5’ss BC mut fwd	TAAGGACTTGTATCATGGACAAGATCAC
	U1-70K 5’ss BC mut rev	GTGATCTTGTCCATGATACAAGTCCTTA
